# Essential oils mouthwash with or without alcohol in relation to effect on parameters of plaque and gingivitis: A systematic review and meta‐analysis

**DOI:** 10.1111/idh.12843

**Published:** 2024-08-12

**Authors:** B. W. M. van Swaaij, G. A. Van der Weijden, R. J. Smith, M. F. Timmerman, D. E. Slot

**Affiliations:** ^1^ Department of Periodontology, Academic Centre for Dentistry Amsterdam (ACTA) University of Amsterdam and Vrije Universiteit Amsterdam Amsterdam The Netherlands; ^2^ Department of Dental Hygiene, Hogeschool Arnhem Nijmegen University of Applied Sciences Nijmegen The Netherlands; ^3^ Department of Implantology and Periodontology Radboud University Medical Center (Radboudumc) Nijmegen The Netherlands

**Keywords:** alcohol, essential oils, gingivitis, mouth rinse, mouthwash, plaque

## Abstract

**Aim:**

The primary aim was to systematically assess the available literature on the effect of an essential oils mouthwash without alcohol (EOalc−) compared to an essential oils mouthwash with alcohol (EOalc+) on plaque scores and parameters of gingival health. The secondary aim was to evaluate user appreciation.

**Materials and Methods:**

The MEDLINE‐PubMed and Cochrane‐CENTRAL databases were searched to identify eligible studies published up to and including March 2024. Papers comparing the effectiveness of EOalc− and EOalc+ were included. The quality was assessed. A descriptive analysis and a meta‐analysis were performed.

**Results:**

After screening, seven papers were found to be eligible. The descriptive analysis demonstrates a significant difference in plaque scores in favour of EOalc+. This is confirmed by the meta‐analyses of plaque scores in non‐brushing and brushing studies (DiffM = 0.40; 95% CI [0.27; 0.53], *p* < 0.00001 and DiffM = 0.05; 95% CI [0.01; 0.10], *p* = 0.01, respectively). This finding is also supported by the sub‐analysis of brands. The meta‐analyses of bleeding and gingival scores in brushing studies did not show significant differences between products. For user appreciation, the difference found was for taste perception in favour of EOalc− (DiffM = 1.63; 95% CI [0.72; 2.55], *p* = 0.0004).

**Conclusion:**

When an EO‐mouthwash is used in non‐brushing or brushing situations, with small to moderate certainty, EOalc− provided less effect regarding plaque control than EOalc+. For bleeding and gingival index there is weak certainty for no difference. In terms of the taste perception EOalc− seems more appreciated.

## 1 INTRODUCTION

Gingivitis and periodontitis are periodontal diseases and are among the most common oral diseases, affecting between 20% and 50% of the global population.^1^ However, some studies show even a higher prevalence of gingivitis. The variation in reported prevalence is often due to sample characteristics and definition criteria.^2^, ^3^, ^4^, ^5^ The high prevalence of periodontal disease is a public health concern. Poor oral hygiene is considered a necessary component cause of periodontal diseases; thus, maintaining oral hygiene plays a key role in the prevention of these diseases.^6^

Inflammation of the periodontal tissues can occur when the dental plaque biofilm is left undisturbed for more than 2 days.^7^ Reduction of plaque by toothbrushing leads to a reduction in gingivitis, which may prevent periodontitis.^8^ Therefore, brushing teeth is recommended as a means of improving and maintaining oral health or preventing oral health diseases.^6^ Interdental cleaning is also associated with reduced interproximal periodontal disease.^9^ Such cleaning can be conducted using products such as dental floss, toothpicks, oral irrigators and interdental brushes.^6^, ^10^

However, mechanical cleaning appears to be difficult, as few people seem to be able to achieve a high level of oral self‐care.^11^ The effectiveness of toothbrushing is affected by multiple factors, including motivation, knowledge and manual dexterity.^8^ When the quality of mechanical plaque control is insufficient, chemical plaque control in the form of a mouthwash (MW) with anti‐microbial ingredients can be considered. The use of MWs can increase user motivation, compliance and the ability to access hard‐to‐reach areas.^12^ MWs are widely appreciated for their ease of use and breath‐freshening effects.^13^ Many brands of MWs with various active agents are available. Based on a meta‐review, it has been proposed that MWs containing chlorhexidine and essential oils (EO) have a large beneficial effect on oral health. This proposal is supported by a strong body of evidence. As chlorhexidine has serious side effects, one of the most reliable long‐term alternatives is an essential oils mouthwash (EO‐MW).^13^

EO‐MWs have been approved by the American Dental Association since 1987 and are globally available. In a fixed EO formula, the combination often comprises thymol (0.064%) and eucalyptol (0.092%) mixed with menthol (0.042%) and methyl salicylate (0.060%). Most EO‐MWs utilize an alcohol‐based vehicle solution. When used as an adjunct to mechanical plaque control, EO‐MWs reduce both plaque and gingivitis.^14^

The alcohol vehicle solution is generally used to both dissolve and stabilize the active ingredients and to improve the product's shelf life. Alcohol also adds to the flavour and provides a ‘strong taste’ to the MW.^12^ However, alcohol‐based MWs are contra‐indicated in patients with mucositis, patients with sensitive tissues due to head and neck radiation therapy, patients sensitive to alcohol, patients with a (former) alcohol addiction and immunocompromised patients.^15^ Therefore, there is a demand for an alcohol‐free EO‐MW (EOalc−) alternative. Moreover, there is an increasing desire for alcohol‐free products for dietary, cultural and religious reasons. As it is the EO and not the alcohol that contributes to the reduction of plaque and gingival inflammation, there is justification for the creation of new formulations without alcohol.^12^ EOalc− products have been on the consumer products market for over 10 years. In a 2‐week experimental gingivitis model, EOalc− was significantly more effective in reducing plaque and gingivitis compared to a 5% hydro‐alcohol MW without EO.^16^ Several clinical studies have evaluated EOalc− compared to EOalc+. However, the effect of an EOalc− product on the parameters of periodontal health has not been systematically evaluated.

Therefore, the purpose of this systematic review was to appraise and synthesize the available scientific literature concerning the efficacy of EOalc− compared to EOalc+ with respect to plaque and gingivitis scores. In addition, user appreciation was evaluated.

## 2 MATERIALS AND METHODS

The preparation and presentation of this systematic review is in accordance with the Cochrane Handbook for Systematic Reviews of Interventions^17^ and the guidelines of the Transparent Reporting of Systematic Reviews and Meta‐Analyses (PRISMA).^18^ The protocol for this systematic review was developed a priori^19^ and registered with the International Prospective Register of Systematic Reviews^20^ under registration number CRD42021224171.

The primary outcome of this study was to evaluate the effect on plaque and gingivitis scores of rinsing with an EOalc− as compared to an EOalc+. The secondary outcome of this study was to evaluate the user appreciation of rinsing with an EO‐MW either with or without alcohol.

### 2.1 Search strategy

A search strategy focusing on the comparison between EOalc− and EOalc+ for the parameters of plaque, bleeding, gingivitis, taste perception, taste alteration and remaining taste after rinsing was created. The search was conducted by two reviewers (BVS and RJS). The National Library of Medicine, Washington D.C. (MEDLINE‐PubMed) and the Cochrane Central Register of Controlled Trials (CENTRAL) were used to find appropriate papers. A systematic electronic search was conducted, including papers published up until March 2024. The search was not limited by year of publication or language. The reference list from each of the included studies was hand‐searched for studies that could be relevant to this systematic review. Furthermore, the following database sources were searched for possible relevant studies that were either unpublished or published in non‐commercial form: (https://opengrey.eu/), the European Federation of Periodontology (http://efp.org) and the International Association for Dental Research (http://www.iadr.org). Manufacturers of EO‐MWs were approached to request any unpublished or ongoing studies that could be considered. Table 1 illustrates the search terms that were used.

**TABLE 1:** Search terms used for Pub Med‐MEDLINE and Cochrane‐CENTRAL.

The following strategy was used in the search:
[< ingredient 1: alcohol >] {<“Alcohols”[Mesh] OR alcohol OR ethanol OR ethylalcohol OR alcohol* OR ethanol*> AND [< ingredient 2: essential oils >] <“tartar control listerine” [Supplementary Concept]) OR “Oils, Volatile”[Mesh] OR LISTERINE OR (essential oils) OR (essential oil) > AND [<product: mouthwash>] < “Mouthwashes” [MeSH] OR Mouthwashes OR Mouthwash OR mouthwash* OR mouthrinses OR mouthrinse OR mouthrins* >}

*Note*: The asterisk (*) was used as a truncation symbol.

### 2.2 Screening and selection

All studies were screened individually and independently by two reviewers (BVS and RJS), initially by title and abstract when accessible. Both reviewers used Rayyan, a web and mobile app for systematic reviews, which is recommended as a suitable and easy‐to‐use tool to support title and abstract screening within healthcare research.^21^ The Rayyan app helps expedite the initial screening of studies by using a process of semi‐automation.^22^ Possible duplicates were identified and checked by the two reviewers to enable the elimination of those that were identical. During the screening process, the reviewers were blinded, so they could not see each other's screening results. Titles and abstracts were categorized as included, excluded or undecided. After the independent screening process, the search was unblinded, and the conflicts that were identified by Rayyan were resolved by the reviewers. Disagreements in the screening and selection process were resolved by consensus or, if disagreement persisted, by arbitration via a third reviewer (DES). Once the list of included titles and abstracts had been created, full‐text versions of the papers were retrieved and screened for suitability. The studies that met all inclusion criteria were further processed to obtain data.

The inclusion criteria included the following:

- Randomized controlled trials (RCT) or controlled clinical trials (CCT)
- Trials conducted with human participants
  - in satisfactory general health (no systemic disorders)
  - aged ≥ 18 years
  - without fixed orthodontic equipment
  - without dental implants
  - without removable dental prosthesis
- Intervention: EOalc−
  Comparison: EOalc+
  (no specific range of duration of MW use was required)
- Outcome parameters relevant to the focus questions:
  - Primary outcomes of interest: plaque, bleeding and gingival inflammation (inclusion of at least one primary outcome was mandatory)
  - Secondary outcomes of interest: user appreciation, such as taste perception, taste alteration and taste remaining after rinsing
- No minimum or limitations in the follow‐up period.

### 2.3 Heterogeneity assessment

Aspects used to determine the clinical heterogeneity of the studies were participant characteristics, groups, variation in alcohol concentration, evaluation period, side effects and industry funding. The diversity of the study designs was used to evaluate methodological heterogeneity. If either the clinical or the methodological heterogeneity was considered to be too high, the source of the heterogeneity was further investigated with a subgroup/sensitivity analysis. The pooling of the results was considered, and statistical heterogeneity was assessed when the participants, interventions and outcomes were sufficiently similar between the individual studies.

### 2.4 Risk of bias assessment

To estimate the risk of bias in each included study, methodological quality was independently assessed by two reviewers (BVS and DES) using a pre‐designed form. Disagreements were resolved by consensus or, if disagreement persisted, by arbitration via a third reviewer (GAW). The Cochrane tools for risk of bias for interventional studies were used: for non‐RCTs, the ROBINS‐I^23^ and for RCTs, the RoB 2.0.^24^ The domains included in RoB 2.0 are bias arising from the randomization process, bias due to deviations from intended interventions, bias due to missing outcome data, bias in measurement of the outcome and bias in selection of the reported result. For crossover RCTs, additional considerations for crossover trials were assessed. For each domain, the tool comprises a series of ‘signalling questions’ that aim to provide a structured approach to eliciting information relevant to an assessment of risk of bias. Based on the answers to the signalling questions, each domain of RoB 2.0 is assigned one of three levels of risk of bias: low, some concerns, or high risk of bias. The overall risk of bias judgement generally corresponds to the highest risk of bias level in any of the domains. However, if a study is judged to have ‘some concerns’ about risk of bias for multiple domains, it is judged as at high risk of bias overall.^24^ To create risk of bias assessment figures, the risk of bias visualization tool (robvis) was used.^25^

### 2.5 Data extraction

The data from the publications that met the selection criteria were extracted and processed for further analysis. As a first step, the original authors, years, details of the study design (such as country of execution, methodology, duration, oral prophylaxes), details on participants (gender, age), groups (brands and regimen) and original conclusion were extracted. As a second step for the heterogeneity analysis, side effects and industry funding were extracted from the included papers. As a third step, data extraction was performed for the primary outcomes of interest (plaque, bleeding and gingival inflammation) and secondary outcomes of interest (user appreciation measures, such as taste perception, taste alteration and taste remaining after rinsing). Two reviewers (BVS and RJS) evaluated the selected publications for mean baseline, end, incremental (difference) scores and standard deviation (SD). To ensure accurate estimates, any data approximation in figures was avoided. In the case of missing data or undetermined information, attempts were made to contact the first or corresponding author of the included publications for clarification or to retrieve additional data.

### 2.6 Data analysis

#### 2.6.1 Descriptive analysis

As a summary of the data, a descriptive data presentation was used for all studies. It was decided a priori to categorize the studies into either monotherapy studies (non‐brushing studies) or studies that also included self‐performed daily oral hygiene (brushing studies). Plaque, bleeding and gingivitis were taken into account. Taste perception, remaining taste after rinsing and taste alteration were considered secondary parameters of user appreciation.

#### 2.6.2 Meta‐analysis

If quantitative methods were feasible, a meta‐analysis was performed to analyse the efficacy of EOalc− compared to EOalc+. The analysis was performed using the Review Manager program (version 5.3) in accordance with the PRISMA guidelines.^18^ A meta‐analysis was only performed if two or more studies could be included.

For outcome parameters that were assessed using the same scoring criteria, a meta‐analysis was performed by calculating the difference of means (DiffM) together with its joined 95% confidence interval (CI). The DiffM between test and control was calculated using both the ‘random and fixed effects’ model where appropriate. It was expected that there would be considerable heterogeneity among the included studies, as study designs and details presumably differ. Random effect models are well suited for meta‐analyses with heterogeneous effects. A fixed effects model was applied when there were fewer than four comparisons^26^ because the estimate of between‐study variance is poor for analyses with low numbers of studies.^17^ Where possible, formal testing for publication bias was used, as proposed by Egger et al.^27^

### 2.7 Assessment of statistical heterogeneity

When a confidence interval has a low level of overlap, it strongly suggests that statistical heterogeneity is present within the studies. The heterogeneity of the included studies was statistically tested using the chi‐squared and *I* ^2^ tests. When chi‐squared tests yielded a p‐value of <0.1, the heterogeneity was considered statistically significant. As an approximate guide to assessing the possible magnitude of inconsistency across studies, the *I* ^2^ statistic was interpreted as follows: 0–40% indicates unimportant levels of heterogeneity, 30–60% represents moderate heterogeneity, 50–90% represents substantial heterogeneity and an *I* ^2^ statistic of >75% indicates considerable heterogeneity. Considerable heterogeneity was evaluated with subgroup and sensitivity analyses to assess effect modification.^28^ Where possible, a sub‐analysis on brands was performed.

### 2.8 Grading the ‘body of evidence’

A modification of the Grading of Recommendations Assessment, Development and Evaluation (GRADE) was used to rank the certainty of the evidence.^29^, ^30^ Two reviewers (BVS and DES) independently rated the quality of the evidence and the strength and direction of the recommendations according to the following aspects: risk of bias, consistency of results, directness of evidence, precision, publication bias and magnitude of the effect. Disagreements between the two reviewers were resolved through additional discussion with a third reviewer (GAW).

## 3 RESULTS

### 3.1 Search and selection results

**FIGURE 1:**
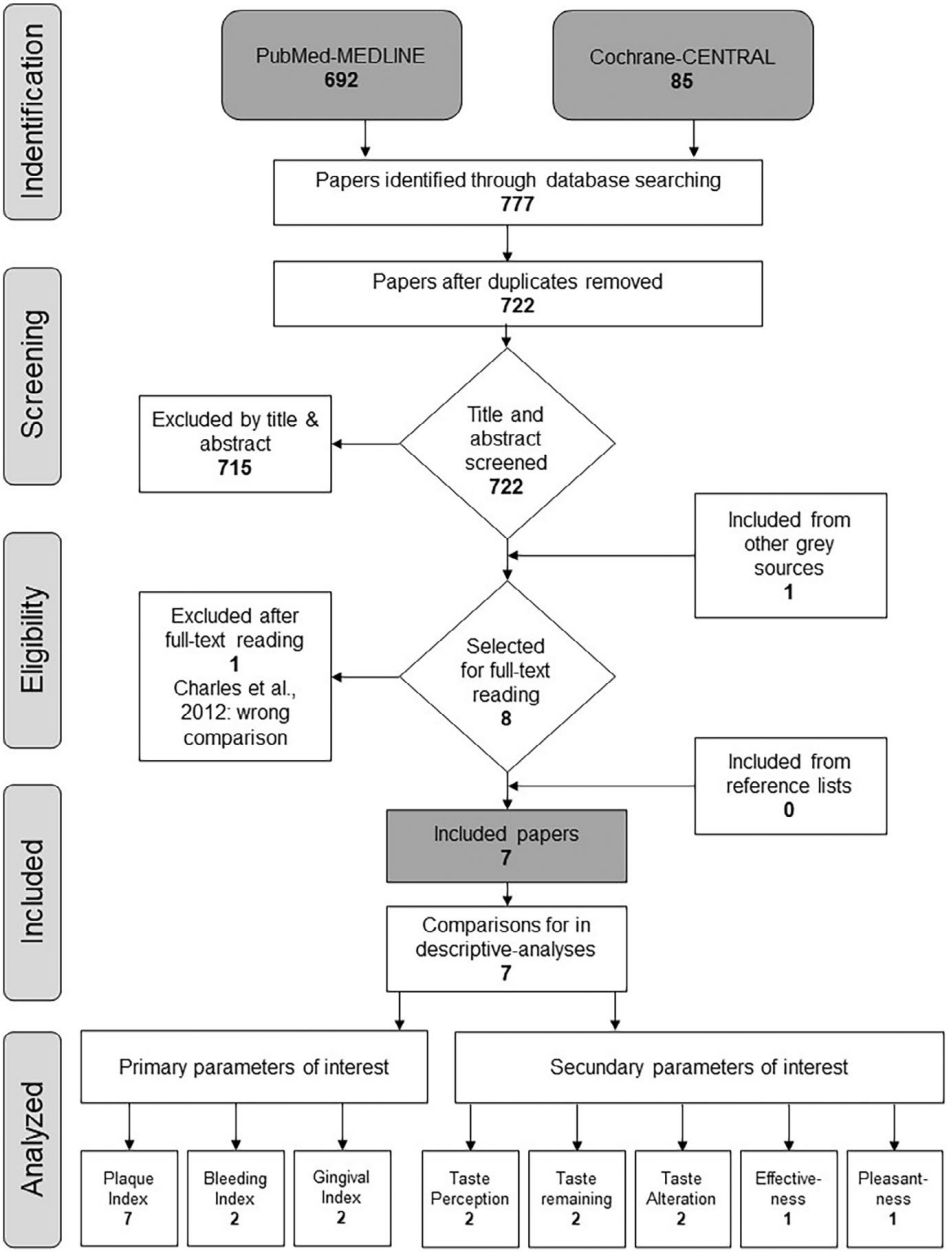
Flowchart.

The search in the PubMed‐MEDLINE and Cochrane‐CENTRAL databases yielded 722 unique studies (Figure 1). The titles and abstracts of these studies were screened, and the reviewers' selections had an overlap of 98.8%. Full texts were obtained for eight potentially eligible studies, of which one study^16^ was excluded after full‐text reading because it included only a comparison of an EO‐MW to a 5% hydro‐alcohol MW without EO and no comparison to an EOalc+. Requests for unpublished data from well‐known companies that produce EO‐MWs (Johnson & Johnson and Curasept) yielded one additional paper written in Italian and published in the grey literature.^31^ Consequently, seven studies (I,^31^ II,^32^ III,^33^ IV,^34^ V,^35^ VI,^36^ VII^37^), including seven comparisons, were included in this systematic review. Four studies (II,^32^ III,^33^ IV,^34^ VI^36^) include a non‐brushing design with a focus on the inhibition of plaque accumulation over a period of 3–4 days. Three studies (I,^31^ VII,^37^ V^35^) are brushing studies that measure both plaque and gingival inflammation over 15 days, 12 weeks and 6 months respectively. All the included studies evaluate one or more of the primary and clinical parameters. Plaque scores were assessed in all studies. Three studies (I,^31^ II,^32^ IV^34^) also investigated the participants' appreciation of the MW, using measures such as taste perception, taste alteration and remaining taste.

### 3.2 Assessment of methodological and clinical heterogeneity

All seven studies were considered to be heterogeneous in methodological design, MW brands and the rinsing procedure used as well as in participant‐related factors such as age and gender. For details, see Table 2.

**TABLE 2:** Overview of the studies processed for data extraction.

# Study authors (year) country, risk of bias	Study design, intervention, duration	Participants base (end), gender, age (mean/range), oral prophylaxis (OP)	Groups brands regimen	Conclusions of the original authors
I. Basso et al. (2010)^31^ Italy Some concerns	RCT Double blinded Crossover 15 days WO: 7 days	31 (28) ♀:? ♂:? Mean age:? Age range:? OP:? Brushing	EOalc− Curasept Daycare; Curaden Healthcare S.r.l., Saronno, Italy EOalc+ Listerine; Johnson & Johnson S.r.l., Italy ?ml for 30 s twice a day TB:? TP:? Instruction: maintain usual oral hygiene	The two EO‐MW were equally effective in reducing oral hygiene indices. The use of the EOalc+ was associated with an increased sensation of ‘burning’. Despite the similar antiplaque activities of these MW, the risk/benefit ratio of daily use of EOalc+ must be examined according to recent scientific evidence
II. Marchetti et al. (2011)^32^ Italy Some concerns	RCT Double blinded Crossover 3 days WO: 14 days, own products	30 ♀: 13 ♂: 17 Mean age: 23.9 Age range: 18–35 OP: Yes Non‐brushing	EOalc− Curasept Daycare, Curaden International AG Kriens, Suisse EOalc+ Listerine®, Johnson & Johnson, S. Palomba‐Pomezia, Italy 20 mL for 60 s twice a day	EOalc− was a less potent plaque inhibitor than the traditional EOalc+. It appears that the subjects appreciated the effect on plaque reduction of the traditional MW better
III. Pizzo et al. (2013)^33^ Italy Some concerns	RCT Double blinded Crossover 4 days WO: 10 days TP: Elmex Protezione Carie TB: Dentosan medio	12 (?) ♀: 4 ♂: 8 Mean age: 25.6 (SD 5.5) Age range: 20–35 OP: Yes Non‐brushing	EOalc− Curasept Daycare; Curaden Healthcare S.r.l., Saronno, Italy EOalc+ Listerine; Johnson & Johnson S.p.A., S. Palomba‐Pomezia, Italy 20 mL for 30 s twice a day	Two EO‐MW, although containing the same active ingredients, did not exert similar plaque inhibition. Moreover, the ethanol‐free rinse was found to inhibit plaque regrowth to the same extent as the saline solution (negative control). These findings suggest that EOalc− would provide poor plaque control benefits when used alongside toothbrushing
IV.Marchetti et al. (2017)^34^ Italy Some concerns	RCT Double blinded Crossover 3 days WO: 14 days, own products	21 ♀: 14 ♂: 7 Mean age: 26.2 Age range: 21–41 OP: yes Non‐brushing	EOalc− Listerine Zero formulation Johnson & Johnson Consumer Inc. EOalc+ Listerine Difesa Denti e Gengive; formulation Johnson & Johnson 15 mL for 60 s twice a day	EOalc− showed the same effect on plaque regrowth as the EOalc+. Due to the short follow‐up, these results could be considered preliminary and we cannot exclude that the tested products could have other effects over the medium or long term
V. Lynch et al. (2018)^35^ Brazil Some concerns	RCT Double blinded Parallel 6 months	247 (230) ♀: 223 ♂: 147 Mean age: 36.4 (SD 13.46) Age range: ≥18–? OP: yes Brushing	EOalc− Listerine Zero; Johnson & Johnson, NJ, USA EOalc+ Listerine Cool Mint; Johnson & Johnson, NJ, USA 20 mL for 30 sec twice a day TB: *REACH®* soft bristled TP: *Colgate®* anti cavity Instruction: brush with one ribbon of TP in usual manner twice a day. Interdental cleaning was allowed if it was part of usual selfcare	EOalc− and EOalc+ were able to reduce plaque, gingivitis and gingival bleeding in comparison to the use of mechanical oral hygiene alone in a 6‐month, randomized study. No significant differences in efficacy in reducing plaque, gingivitis and gingival bleeding were found between EOalc− and EOalc+ formulations
VI. Spuldaro et al. (2021)^36^ Brazil Some concerns	RCT Double blinded Crossover 4 days WO: 10 days, own products	11 (11) ♀: 6 ♂: 5 Mean age: 21.1 (SD 3.67) Age range: 18–40 OP: yes Non‐brushing	EOalc− Listerine Zero; Johnson & Johnson, Sao Paulo, Brazil EOalc+ Listerine Cool Mint; Johnson & Johnson, Sao Paulo, Brazil 20 mL for 30 s twice a day	EOalc+ presented better results in retarding the early supragingival and subgingival plaque formation compared with EOalc−
VII. Bosma et al. (2024)^37^ USA Some concerns	RCT Single blind Parallel 12 weeks	108 (102) ♀: 85 ♂: 23 Mean age: 43.8 Age range:? OP: yes Brushing	EOalc− Listerine Cool Mint Zero; Johnson & Johnson, NJ, USA EOalc+ Listerine Cool Mint; Johnson & Johnson, NJ, USA 20 mL for 30 s twice a day	Twice daily use of EOalc+ or EOalc− in conjunction with manual toothbrushing significantly reduced plaque, gingivitis and bleeding at 4 and 12 weeks, compared to brushing alone

Abbreviations: Alc−, non‐alcoholic; Alc+, containing alcohol; EO, essential oils; MW, mouthwash; TB, toothbrush; TP, toothpaste; WO, wash out.

All seven studies were designed as RCTs, of which five used a crossover design (I,^31^ II,^32^ III,^33^ IV,^34^ VI^36^) and two (V,^35^ VII^37^) used a parallel design. A washout period was allowed for 14 days (II,^32^ IV^34^), 10 days (III,^33^ VI^36^) or 7 days (I^31^). Participants were prohibited from rinsing with water after rinsing with their assigned product (II,^32^ IV^36^). Study III^33^ restricted the participants from rinsing, eating or drinking within the first hour after rinsing, and participants were instructed to not use any other rinse or chewing gum. The participants in study VI^36^ were told not to eat or drink anything for at least 30 min after rinsing. In study VII,^37^ participants were asked to refrain from the use of probiotic drinks/supplements for 1 week prior to and throughout the study and to abstain from chemotherapeutic antiplaque/anti‐gingivitis products for 4 weeks prior to the start. In addition, they were not allowed to eat for at least 4 h before the assessments. Study I^31^ did not include participants who were using chewing gum on a regular basis. Oral prophylaxis was conducted at the beginning of the experimental phase in the majority of the studies (II,^32^ III,^33^ IV,^34^ V,^35^ VI,^36^ VII^37^). It is unclear whether oral prophylaxis was performed at the beginning of each phase of study I.^31^

The number of participants in five studies was between 11 and 30, and two studies (V,^35^ VII^37^) included over 100 participants. The participants in the experiments for study IV^34^ and study VI^36^ were described as dental or dental hygiene students, whereas one study (II^32^) specifically mentioned that the participants were not dental students. Participants for study III^33^ were selected based on having ≥22 natural teeth with two scorable surfaces. Other inclusion criteria included having 20 or more evaluable teeth (II,^32^ IV,^34^ VI^36^), a modified gingival index of ≥1.95 (V^35^), a Turesky modification of the Quigley‐Hein plaque index of ≥1.95 (V^35^), no clinically identified gingivitis and/or periodontitis (VI^36^), no severe periodontitis with a probing depth of ≥5 mm and/or attachment loss of ≥2 mm (II,^32^ IV^34^) and no periodontitis according to Tanner's definition^38^ of early periodontitis (V^35^).

With regard to MW brands used, the intervention group for four studies (IV,^34^ V,^35^ VI,^36^ VII^37^) used Listerine Zero, which is an EOalc−. In three studies (I,^31^ II,^32^ III^33^), the intervention group used Curasept Daycare, which is also an EOalc−. All the control groups received an EOalc+ of Listerine of different formulations with a concentration of 21.3% (II,^32^ IV^34^), 21.6% (III^33^), or 22% ethanol (I^31^). Ethanol concentration was not reported in studies V,^35^ VI^36^ and VII.^37^ In most studies, the rinsing was conducted without supervision; only the first rinsing procedure was supervised in two studies (III,^33^ V^35^). In study VII,^37^ the first procedure was conducted under supervision in the clinic, then on weekday mornings under virtual supervision via video call.

User appreciation was evaluated with the aid of a visual analogue scale (II,^32^ IV^34^) and a questionnaire (I^31^). No side effects were reported or observed in three studies (II,^32^ IV,^34^ VI^36^). The use of EOalc+ was associated with an increased sensation of ‘burning’ (I^31^), and one participant reported experiencing dyspepsia (V^35^). Dental hypersensitivity was reported by a participant twice (III,^33^ V^35^) for EOalc+ and once for EOalc− (V^35^). Oral mucosal exfoliation was experienced by four participants in the EOalc+ group and four participants in the EOalc− group (VII^37^). The authors of five studies declared no conflict of interest (I,^31^ II,^32^ III,^33^ IV,^34^ V,^35^). Studies V^35^ and VII^37^ includes the disclaimer that the majority of the authors were employees of Johnson & Johnson, which also funded the study. The authors of study IV^34^ explicitly state that they self‐financed the study and that the companies GABA Vebas and Johnson & Johnson Italia only provided the study products. Curaden Healthcare supplied the products for study I.^31^ The authors of study VI^36^ mention that they did not receive any specific grant from funding agencies in the public, commercial, or not‐for‐profit sectors.

### 3.3 Risk of bias assessment

The estimated potential risk of bias in the included studies was assessed (for details, see Appendix S1). All studies were deemed to have a low risk of bias in the applicable domains of randomization process, period and carry‐over effects, missing outcome data and measurement of the outcome. All of the studies were scored as having some concerns regarding the selection of reported results. Only four studies (II,^32^ IV,^34^ V,^35^ VII^37^) published an online trial protocol but did not include a statistical analysis plan and therefore received this judgement. In six studies, there were some concerns about bias due to deviations from intended interventions because there was no information about the analysis used to estimate the effect of assignment to intervention (I,^31^ II,^32^ III,^33^ IV,^34^ VI,^36^ VII^37^). Only study V^35^ specifically reported the Intent‐to‐Treat principle. The overall risk of bias was estimated to be ‘some concerns’ for all studies.

### 3.4 Study outcome results

Appendix S2 presents the results of the data extraction for all seven studies. A description of each parameter and index is provided. If applicable, the baseline scores, end scores and the incremental difference between baseline and end are included.

#### 3.4.1 Descriptive analysis

Table 3 illustrates the statistical differences as a summary of findings between EOalc− and EOalc+ for all parameters of interest in this systematic review. The descriptive analysis demonstrates that three non‐brushing studies (II,^32^ III,^33^ VI^36^) found a significant difference in plaque scores in favour of EOalc+. Two out of three brushing studies did not find a difference. For the other clinical parameters, no significant differences were found. Regarding the secondary parameters of interest, significant differences in favour of EOalc− were observed, once for taste perception (IV^34^) and once for pleasantness of taste (I^31^).

**TABLE 3:** A descriptive summary of statistical significance levels of the use of mouthwashes with or without alcohol on the parameters of interest.

			Clinical parameters			User appreciation					
Author, year	Design	Intervention	PI	BI	GI	TP	TR	TA	P	E	Control
Marchetti et al. (2011)^32^	NB	EOalc− (CS)	‐	□	□	○	○	○	□	□	EOalc+
Pizzo et al. (2013)^33^	NB	EOalc− (CS)	‐	□	□	□	□	□	□	□	EOalc+
Marchetti et al. (2017)^34^	NB	EOalc− (LZ)	○	□	□	+	○	○	□	□	EOalc+
Spuldaro et al. (2021)^36^	NB	EOalc− (LZ)	‐	□	□	□	□	□	□	□	EOalc+
Basso et al. (2010)^31^	B	EOalc− (CS)	○	○	□	?	□	?	+	○	EOalc+
Lynch et al. (2018)^35^	B	EOalc− (LZ)	○	○	○	□	□	□	□	□	EOalc+
Bosma et al. (2024)^37^	B	EOalc− (LZ)	–#	?	○#	□	□	□	□	□	EOalc+
	Total		3/7 ○ 4/7 −	2/2 × ○	2 × ○	1/2 ○	2/2 ○	2/2 ○	1× +	1× ○	

Abbreviations: Alc‐, Without Alcohol; Alc+, With Alcohol; BI, Bleeding Index; CS, Curasept; E, Effectiveness; GI, Gingival Index; LZ, Listerine Zero; MW, Mouthwash; NB, Non Brushing; B, Brushing; P, pleasantness; PI, Plaque Index; TA, Taste alteration; TP, Taste perception; TR, Taste remaining; #, adapted from the meta‐analysis performed (Appendix S4, S6).

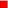
 − Significant difference in favour of comparison; EOalc+.

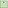
 + Significant difference in favour of intervention; EOalc−.

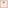
 ○ No significant difference.

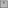
 □ No data available (not tested).

#### 3.4.2 Meta‐analysis

For the primary parameters of interest, namely plaque, bleeding and gingivitis scores, it was possible to perform a meta‐analysis (Tables 4a, 4b, 4c, 4d). All except two (I,^31^ VI^36^) of the included studies evaluated plaque scores using the Turesky modification^39^ of the Quigley‐Hein plaque index.^40^

**TABLE 4a:** Meta‐analysis for data evaluating *non‐brushing studies* on the efficacy of EOalc− compared to EOalc+ on primary parameter plaque scores.

Index	Measurement moment	Included studies	Model	DiffM	Test overall		Test for heterogeneity		
					95% CI	*p*‐value	*I* ^2^ value (%)	*p*‐value	For details see Appendix
Plaque Index Non‐brushing *Turesky modification* ^39^ *of the Quigley‐Hein Plaque Index* ^40^	End	Marchetti et al. (2011) Marchetti et al. (2017) Pizzo et al. (2013)	Fixed	0.40	[0.27; 0.53]	<0.00001	96%	<0.00001	S3a
Plaque Index Non‐brushing Sub‐analysis Curasept *Turesky modification* ^39^ *of the Quigley‐Hein Plaque Index* ^40^	End	Marchetti et al. (2011) Pizzo et al. (2013)	Fixed	0.61	[0.44; 0.78]	<0.00001	97%	<0.00001	S3b

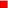
 Significant difference in favour of comparison; EOalc+.

**TABLE 4b:** Meta‐analysis for data evaluating *brushing studies* on the efficacy of EOalc− compared to EOalc+ on primary parameter plaque index.

Index	Measurement moment	Included studies	Model	DiffM	Test overall		Test for heterogeneity		
					95% CI	*p*‐value	*I* ^2^ value (%)	*p*‐value	For details see Appendix
Plaque Index Brushing *Turesky modification* ^39^ *of the Quigley‐Hein Plaque Index* ^40^	Base	Lynch et al. (2018) Bosma et al. (2024)	Fixed	−0.02	[−0.08; 0.04]	0.51	0%	<0.00001	S4a
Plaque Index Brushing *Turesky modification* ^39^ *of the Quigley‐Hein Plaque Index* ^40^	End	Lynch et al. (2018) Bosma et al. (2024)	Fixed	0.05	[0.01; 0.10]	0.01	95%	<0.00001	S4b

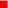
 Significant difference in favour of comparison; EOalc+.

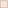
 No significant difference.

**TABLE 4c:** Meta‐analysis for data evaluating *brushing studies* on the efficacy of EOalc− compared to EOalc+ on primary parameter bleeding index.

Index	Measurement moment	Included studies	Model	DiffM	Test overall		Test for heterogeneity		
					95% CI	*p*‐value	*I* ^2^ value (%)	*p*‐value	For details see Appendix
Bleeding index Ainamo and Bay^74^	Base	Basso et al. (2010) Lynch et al. (2018)	Fixed	−0.00	[−0.04; 0.03]	0.81	0%	0.94	S5a
Bleeding index Ainamo and Bay^74^	End	Basso et al. (2010) Lynch et al. (2018)	Fixed	−0.01	[−0.02; 0.01]	0.47	0%	0.81	S5b
Bleeding index Ainamo and Bay^74^	Diff	Basso et al. (2010) Lynch et al. (2018)	Fixed	0.00	[−0.04; 0.04]	0.99	0%	0.83	S5c

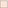
 No significant difference.

**TABLE 4d:** Meta‐analysis for data evaluating *brushing studies* on the efficacy of EOalc− compared to EOalc+ on primary parameter gingival index.

Index	Measurement moment	Included studies	Model	DiffM	Test overall		Test for heterogeneity		
					95% CI	p‐value	*I* ^2^ value (%)	p‐value	For details see Appendix
*Modified Gingival Index* *Lobene* ^75^	Base	Lynch et al. (2018) Bosma (2024)	Fixed	−0.00	[−0.04; 0.03]	0.93	61%	0.11	S6a
*Modified Gingival Index* *Lobene* ^75^	End	Lynch et al. (2018) Bosma (2024)	Fixed	0.03	[−0.00; 0.07]	0.07	0%	0.73	S6b

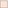
 No significant difference.

The meta‐analysis of plaque scores used in a non‐brushing model showed a significant difference (DiffM = 0.40; 95% CI [0.27; 0.53], *p* < 0.00001) in favour of EOalc+ (Table 4a, Appendix S3a). This result is supported by the sub‐analysis of non‐brushing studies using Curacept Daycare (p < 0.00001) (Table 4a, Appendix S3b). The meta‐analysis of plaque scores in a brushing model also showed a significant difference in favour of EOalc+ (DiffM = 0.05; 95% CI [0.01; 0.10], *p* < 0.01), see Table 4b, Appendix S4b.

The meta‐analyses of bleeding index and gingival index did not show significant differences between EOalc− and EOalc+ products (Tables 4c, 4d, Appendix S5, S6). Regarding the secondary parameters of interest, three meta‐analyses could be performed on taste perception, remaining taste after rinsing and alteration of taste (Table 4e, Appendix S7–S9). A significant difference in favour of EOalc− was found for taste perception (DiffM = 1.63; 95% CI [0.72; 2.55], *p* = 0.0004).

**TABLE 4e:** Meta‐analysis for end data evaluating *non‐brushing studies* on the efficacy of EOalc− compared to EOalc+ on secondary parameters; taste perception, taste remaining and taste alteration.

Index	Measurement moment	Included studies	Model	DiffM	Test overall		Test for heterogeneity		
					95% CI	*p*‐value	*I* ^2^ value (%)	*p*‐value	For details see Appendix
*Taste Perception* *Visual Analogue Scale*	End	Marchetti et al. (2011) Marchetti et al. (2017)	Fixed	1.63	[0.72; 2.55]	0.0004	0%	0.65	S7
*Taste Remaining* *Visual Analogue scale*	End	Marchetti et al. (2011) Marchetti et al. (2017)	Fixed	−0.27	[−1.12; 0.59]	0.54	0%	0.50	S8
*Taste Alteration* *Visual Analogue Scale*	End	Marchetti et al. (2011) Marchetti et al. (2017)	Fixed	0.56	[−0.38; 1.50]	0.24	0%	0.87	S9

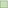
 Significant difference in favour of intervention; EOalc−.

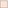
 No significant difference.

Publication bias could not be tested because fewer than 10 studies were included in the meta‐analysis, which would result in insufficient statistical power.^17^, ^27^ Consequently, publication bias cannot be ruled out.

### 3.5 Evidence profile

A summary of the criteria used to rate the quality of evidence is presented in Table 5. Strength of recommendation was assessed as proposed by Guyatt et al.^30^ and Moher et al.^41^ Overall, it was determined that when an EO‐MW is used in non‐brushing or brushing situations, there is small to moderate certainty that EOalc− is less effective in controlling plaque than EOalc+. With regard to bleeding and gingivitis scores there is weak evidence there was no difference between EOalc+ and EOalc−. With respect to user appreciation, in the majority of studies, no difference could be found. In terms of taste perception, users seem to prefer EOalc−.

**TABLE 5:** Summary of findings based on descriptive and meta‐analysis on the quality and body of evidence on the estimated evidence profile^76^ and appraisal of the strength of the recommendation regarding the efficacy of EOalc− on the parameters of interest.

	Primary outcome			Secondary outcome
	Plaque	Bleeding	Gingival index	User appreciation
Non‐brushing/brushing	NB/B	B	B	NB
# experiments descriptives analysis (Table 3)	7	2	2	3
# experiments in Meta‐analysis (Table 4a)	5	2	2	2
Risk of bias (Appendix S1)	Some concerns	Some concerns	Some concerns	Some concerns
Consistency	Rather consistent	Rather consistent	Rather consistent	Rather consistent
Directness	Generally direct	Generally direct	Generally direct	Generally direct
Precision	Rather precise	Rather precise	Rather precise	Rather precise
Reporting bias	Possible	Possible	Possible	Possible
Magnitude of the effect (Tables 3 and 4a)	NB: small against	None	None	None—very small
	B: small against			
Strength and direction of the recommendation	NB: Moderately against	Weak no difference	Weak no difference	Very weak in favour
	B: Small against			
Overall recommendation	When an EO‐MW is used in non‐brushing or brushing situations, with small‐to‐moderate certainty, EOalc− provided less effect regarding plaque control than EOalc+. For bleeding and gingival index, there is weak certainty for no difference. With regard to user appreciation in majority no difference could be found. In terms of taste perception, EOalc− seems more appreciated			

Abbreviations: B, brushing; NA, not applicable; NB, non‐b‐rushing.

## 4 DISCUSSION

The primary aim of this systematic review was to investigate the effect of EOalc− and EOalc+ on plaque scores and parameters of gingival health. To synthesize the data, a structured evaluation was performed in the form of descriptive and meta‐analyses. In the descriptive analysis, three non‐brushing studies (I,^31^ III,^33^ VI^36^) and one brushing study (VII^37^) indicated a significant difference in plaque scores in favour of EOalc+. Both the meta‐analyses, based on non‐brushing studies and the meta‐analysis based on brushing studies support this finding; showing a significant difference in favour of EOalc+ on plaque scores (*p* < 0.00001 and *p* = 0.01 respectively). This result is also supported by the sub‐analysis of non‐brushing studies that used Curacept Daycare (*p* < 0.00001). For gingival health, no difference could be found in any of the analyses of the present review.

### 4.1 User appreciation

The addition of alcohol to MW solutions serves the purposes of preservation and flavouring. It provides a ‘strong taste perception’ to the MW.^12^ Therefore, the secondary parameters of interest in the present systematic review related to participants' appreciation. Only three studies evaluated this aspect.^31^, ^32^, ^34^ The meta‐analysis (Table 4e) based on two studies (II,^32^ IV^34^) that examined remaining taste and alteration of taste did not find a difference between EOalc− and EOalc+. However, a significant difference in favour of EOalc− was found for taste perception (Table 4e). These results align with those of a recent study on the gustatory perception of EO, which showed that EOalc− is perceived as better tasting than EOalc+.^42^ In study III,^33^ the burning intensity was significantly higher for EOalc+, which could imply that alcohol would negatively influence taste perception. This burning intensity could also have affected participants' blinding to the study products. Although all included studies were reported to be double‐blind, some of the participants may have discovered their group assignment by recognizing the traditional EO‐MW taste. EOs themselves can impact taste and contribute to the taste perception or tolerability profile. Moreover, it is likely that an alcohol substitute ingredient, such as propylene glycol or polymer 407 (used for the purpose of solubilizing the EO), would also impact taste perception.

### 4.2 ADA guideline on chemotherapeutic products

This is the first meta‐analysis comparing the effects of EOalc− and EOalc+. Unfortunately, 11 out of 12 meta‐analyses are based on just two comparisons, as the different parameters of interest were measured using several scoring criteria or indices across studies. In particular, participants' preferences were not evaluated structurally in the same manner. This inconsistency in methodology is a call for action to establish a standard manner of evaluating the user appreciation of oral care products. Still, the ADA guideline on Chemotherapeutic Products for Control of Gingivitis^43^ does not require an analysis of participants' appreciation of a product. This by itself is remarkable for a seal of approval that is recognized by dental care professionals and consumers. The requirements mainly focus on evaluating safety, adverse events and efficacy. As taste perception can strongly influence patient compliance, in particular, for a long‐term‐use product such as an EO‐MW, future studies should address this aspect. The ADA guidelines^43^ require evidence of subject compliance, and most clinical studies accomplish this goal by measuring product weights and volumes at baseline and post‐treatment visits as well as through patients' records of their daily use of the assigned product. However, compliance in a research setting does not always translate directly to daily clinical use. Participating in a clinical study with a potential reward for following the proposed protocol is different from receiving a clinical recommendation from a dental care professional.

The ADA Acceptance Program accepts both crossover‐ and parallel‐design studies. For a crossover design, they recommend the inclusion of an adequate latent period between study periods because of a possible retained effect of some agents.^43^ This is the reason for ongoing discussion in the research community as to whether crossover studies are appropriate for evaluating chemical oral hygiene agents and, if so, how long a washout for MWs should be. The wash‐out periods of the included crossover studies ranged from 7 to 14 days (for details, see Table 2). In the literature, wash‐out periods for MWs range from 3 days to 10 days.^44^ There is a need for adjustment in the guidelines because, at present, no minimum wash‐out period can be justified. In addition, this systematic review included four non‐brushing studies,^32^, ^33^, ^34^, ^36^ which are a research model for evaluating a product as a proof of principle. It is, however, generally unreasonable to use an MW as a replacement for daily oral hygiene except in specific cases, such as following oral surgery.

### 4.3 Essential oils

EO‐MWs were initially marketed and commonly known as Listerine®, with a fixed formula containing the EOs thymol (0.06%), eucalyptol (0.09%), menthol (0.04%) and methyl salicylate (0.05%), with either 21.6% or 26.9% hydro‐alcohol as a vehicle solution.^45^ There are several variants of this brand of EO, which differ in and purpose of treatment. All of the studies included in this systematic review used EOalc+ of the Listerine brand as a control. As an intervention, the EOalc− of two brands were used, which were Listerine Zero (IV,^34^ V,^35^ VI,^36^ VII^37^) and Curasept Daycare (I,^31^ II,^32^ III^33^). These brands both include the four EOs thymol, methyl salicylate, menthol and eucalyptol. These compounds have been evaluated for their effectiveness in addressing supragingival plaque and gingivitis.^14^ Although these ingredients are identical, there are formulation differences, primarily in the ingredients added for preservation, stability, flavour and therapeutic reasons. This difference in composition may explain the reduced effect of Curasept Daycare compared to the traditional EOalc+, Listerine, that was found in two out of three comparisons. Listerine is the leading MW in the United States and is globally available.^46^ Unfortunately, due to its cost, it is inaccessible to patients in third‐world countries with a limited budget.^47^ *Lippia sidoides*, of the Verbenaceae family, is a low‐cost medicinal herb that has been used in traditional Brazilian medicine for a variety of antifungal and antimicrobial purposes.^48^ Biochemical data have shown that oils and extracts from *Lippia sidoides* are rich in thymol and carvacrol compounds, which are also known for their antimicrobial properties. This herbal plant is popularly known as ‘alecrim pimenta’ and is widely available in northeast Brazil.^48^ Previously, an MW prepared using *Lippia sidoides* EO was applied to the teeth of German Shepherd dogs with marginal gingivitis every 2 days for 2 weeks, and significant reductions in plaque and gingivitis scores were found.^49^ Two studies^47^, ^48^ evaluated the efficacy of a self‐prepared *Lippia sidoides* EO‐MW in humans and demonstrated that it is effective in reducing plaque and gingival inflammation. These findings suggest that EO can be an effective ingredient for improving oral health, as in these *Lippia sidoides* EO‐MW formulations, no alcohol base vehicle was used. However, long‐term studies are needed to confirm the findings, particularly regarding gingival health.

### 4.4 Alcohol

Alcohol is present in many MWs and is often added as a preservative.^50^ In general, it is used to both dissolve and stabilize certain active ingredients and to improve the product's shelf life.^51^ Concerns have been raised regarding the concentration of alcohol in MWs in relation to the risk of oropharyngeal cancer. For many years, dental care professionals, patients and researchers have been discussing the safety of alcohol‐containing MWs for daily use. Many narrative reviews and several correlation studies have been published.^52^, ^53^, ^54^ Approximately 10 years ago, a critical review of data published over three decades showed that a link between use of MWs, specifically MWs with alcohol, and oral cancers is not supported by epidemiological evidence.^55^ A quantitative analysis based on a systematic review of MW use and oral malignancy also showed no association between the use of MW specifically containing alcohol and the risk of oral cancer.^56^ Moreover, a very recent systematic review found that it cannot be concluded that the use of MW represents an independent risk factor for the development of head and neck cancer.^57^ However, the cancer risk may increase when use of MW occurs in association with other carcinogenic risk factors, such as tobacco and alcohol consumption.^57^, ^58^ Consequently, the likelihood of developing oral cancer appears to be significantly affected by lifestyle choices. Furthermore, it is important to note that human papillomavirus (HPV) has been attributed to oropharyngeal cancers because HPV‐associated oropharyngeal squamous cell carcinoma (OPSCC) comprises approximately 25% of all head and neck cancers.^59^ Epidemiologic studies have shown an increase of HPV‐OPSCC in recent decades, whereas tobacco‐related head and neck cancer rates are decreasing worldwide.^60^

Another aspect of alcohol that is debated is its effect on composite dental restorations. Several studies^61^, ^62^, ^63^, ^64^ evaluated the effect of alcohol‐containing MWs on composites. Two studies^61^, ^62^ concluded that the sorption and solubility of composites were higher in alcohol‐containing rinses. Both preferred alcohol‐free MWs in patients with extensive restorations. It is also mentioned that alcohol‐containing MWs with a low pH may increase sorption and solubility.^63^ In contrast, it has also been shown that long‐term exposure to alcohol‐containing, low pH (<5.5) MWs caused no ultra‐structural and biochemical changes in human enamel and restorative materials.^64^ In summary, the effects of alcohol‐containing MWs on restorations are contradictory, and all studies have the limitation of employing in‐vitro designs. Therefore, the results should be carefully interpreted for clinical premises.

### 4.5 Limitations

‐ Few studies measured multiple parameters of interest. Plaque was measured in all studies; however, only five studies used the same plaque index. Bleeding and gingivitis were measured in two studies. Three out of seven included studies assessed user appreciation. More clinical trials are needed to generate a complete overview of the effect of EOs on gingival health.

‐ Most studies included small sample sizes. The ADA guideline on Chemotherapeutic Products for Control of Gingivitis^43^ requires a minimum sample size of 30 subjects; this minimum was not met by four out of seven studies. There is a need for studies with increased power to detect potential differences.

‐ The included studies differed significantly in study length, which ranged from 3 days to 6 months. The ADA Acceptance Program states that crossover designs may not be practical in the long‐term studies required to adequately evaluate product efficacy. In general, a minimum study duration of 3 months is needed to assess the effect of MWs on gingivitis.^43^ Only two (VII,^37^ V^35^) of the included studies had a sufficient evaluation time (3 months and 6 months, respectively). Long‐term clinical trials are usually at least 6 months in duration to provide clinically significant and meaningful additional benefits for the traditional EOalc+ in reducing plaque and gingivitis as an adjunct to usual oral hygiene.^65^, ^66^, ^67^, ^68^, ^69^, ^70^, ^71^ Long‐term studies are more realistic, as participants also need to continue with daily brushing and use the MW as an adjunct. As the present review included only one long‐term clinical trial (V^35^), only very weak evidence emerges supporting the absence of a difference between EOalc− and EOalc+.

‐ Although methodological and clinical heterogeneity was assessed and the risk of bias was estimated, the validity of some study aspects is a concern. In non‐brushing crossover studies^31^ that do not include an oral prophylaxis at the beginning of each phase and do not take into account baseline values in the statistical analysis, subjects could have been unbalanced for plaque and bleeding on the first day of each test period. Formulating an oral hygiene product is complex, and ingredient differences might impact efficacy, stability and bioavailability, which can create subject bias in an efficacy study. Therefore, repackaging commercial formulations to maintain double blinding can only be done carefully by a validated good manufacturing practices process to maintain the stability and bioavailability of the product and avoid adulteration. These aspects are not a standard point for the bias assessment; however, they may be considered as serious methodological design flaws.

### 4.6 Recommendation

It is suggested that EO‐MWs should be considered the first choice for daily use as adjuvants to self‐mechanical plaque control. At present, based on the data presented in systematic reviews,^12^, ^14^, ^68^, ^69^, ^70^, ^71^ EO‐MWs can be considered the gold standard for daily home use. Additional brushing studies evaluating both EOalc− and EOalc+ are needed to determine the benefits for both plaque and gingival inflammation. In particular, the research design aspects from the ADA guideline on Chemotherapeutic Products for Control of Gingivitis should be taken into account. However, the strong taste of EO could be a limitation for some patients.^72^ As this limitation may be partly due to the alcohol vehicle solution, EOalc− products are of interest. EOalc− MWs were associated with improved taste perception. Further studies with a focus on participants' preferences are indicated. An EOalc− may also be more desirable for paediatric populations.^73^

## 5 CONCLUSION

When an EO‐MW is used in non‐brushing or brushing situations, with small to moderate certainty, EOalc− provided less effect regarding plaque control than EOalc+. For bleeding and gingival index, there is weak certainty for no difference. In terms of the taste perception EOalc− seems more appreciated.

## 6 CLINICAL RELEVANCE

### 6.1 Scientific rationale for the study

Several mouthwashes (MWs) on the global market contain essential oils (EO) and use alcohol as a vehicle solution. This combination has been proven to be effective. Lately, new EO alcohol‐free formulas (EOalc−) have been developed. For various reasons, there is increasing interest in alc‐ MWs. The effect of EOalc− is unknown.

### 6.2 Principal findings

Essential oils mouthwash without alcohol was less effective in plaque control than EOalc+. There was no difference between EOalc+ and EOalc− with regard to bleeding and gingivitis scores. EOalc− scored better on taste appreciation.

### 6.3 Practical implications

There is moderate certainty that both EOalc+ and EOalc− can be recommended as adjuncts to toothbrushing. An EOalc− can be considered a valid recommendation due to its perceived better taste, which may improve patient compliance.

## AUTHOR CONTRIBUTIONS

BvS: contributed to conception, search and selection and design, analysis and interpretation, drafted and critically revised the manuscript. GAW: contributed to conception and design, analysis and interpretation, drafted and critically revised the manuscript. RJS: contributed to search and selection, analysis and interpretation and prepared a preliminary draft of the manuscript. MFT: contributed to analysis and interpretation and critically revised the manuscript. DES: contributed to conception and design, search and selection, analysis and interpretation and critically revised the manuscript. All authors gave final approval and agreed to be accountable for all aspects of work ensuring integrity and accuracy.

## FUNDING INFORMATION

This research received no specific grant from any funding agency in the public, commercial or not‐for‐profit sectors. For this study, no funding was accepted, except for support from the listed institutions. The Dutch Research Council NWO (Nederlandse Organisatie voor Wetenschappelijk Onderzoek) funds the PhD position of the first author.

## CONFLICT OF INTEREST STATEMENT

Van Swaaij and Timmerman declare no conflicts of interest. Van der Weijden, Slot and their research team at ACTA have previously received either external advisor fees, lecturer fees or research grants from dental care product manufacturers. Those manufacturers included GABA/Colgate, Dentaid, Lactona, Oral‐B/Procter & Gamble, Sara Lee, Sunstar Philips Unilever, GSK, Listerine and Waterpik.

## ETHICS STATEMENT

Ethical approval was not required, the protocol was registered at PROSPERO by CRD42021224171.

## Supporting information


Appendix S1–S10.

## Data Availability

Data sharing is not applicable to this article as no new data were created or analysed in this study.

## References

[1] Nazir MA . Prevalence of periodontal disease, its association with systemic diseases and prevention. Int J Health Sci. 2017;11(2):72‐80. PMC5426403 28539867

[2] Idrees MM , Azzeghaiby SN , Hammad MM , Kujan OB . Prevalence and severity of plaque‐induced gingivitis in a Saudi adult population. Saudi Med J. 2014;35(11):1373‐1377. 25399215 PMC4362151

[3] Carvajal P , Gomez M , Gomes S , et al. Prevalence, severity, and risk indicators of gingival inflammation in a multi‐center study on south American adults: a cross sectional study. J Appl Oral Sci. 2016;24(5):524‐534. 27812624 10.1590/1678-775720160178 PMC5083031

[4] Murillo G , Vargas MA , Castillo J , et al. Prevalence and severity of plaque‐induced gingivitis in three Latin American cities: Mexico City‐Mexico, great metropolitan area‐Costa Rica and Bogota‐Colombia. Int J Dent Sc. 2018;20(2):91‐102.

[5] Mostafa B , El‐Refai I . Prevalence of plaque‐induced gingivitis in a sample of the adult Egyptian population. Open Access Maced J Med Sci. 2018;6(3):554‐558. 29610619 10.3889/oamjms.2018.131 PMC5874384

[6] Chapple IL , Van der Weijden F , Doerfer C , et al. Primary prevention of periodontitis: managing gingivitis. J Clin Periodontol. 2015;42(Suppl 16):S71‐S76. 25639826 10.1111/jcpe.12366

[7] Axelsson PA . Commentary: periodontitis is preventable. J Periodontol. 2014;85(10):1303‐1307. 25255150 10.1902/jop.2014.140336

[8] Yaacob M , Worthington HV , Deacon SA , et al. Powered versus manual toothbrushing for oral health. Cochrane Database Syst Rev. 2014;2014(6):CD002281. 24934383 10.1002/14651858.CD002281.pub3 PMC7133541

[9] Marchesan JT , Morelli T , Moss K , et al. Interdental cleaning is associated with decreased Oral disease prevalence. J Dent Res. 2018;97(7):773‐778. 29481764 10.1177/0022034518759915 PMC6728587

[10] Husseini A , Slot DE , Van der Weijden GA . The efficacy of oral irrigation in addition to a toothbrush on plaque and the clinical parameters of periodontal inflammation: a systematic review. Int J Dent Hyg. 2008;6(4):304‐314. 19138181 10.1111/j.1601-5037.2008.00343.x

[11] Petersen PE , Bourgeois D , Ogawa H , Estupinan‐Day S , Ndiaye C . The global burden of oral diseases and risks to oral health. Bull World Health Organ. 2005;83(9):661‐669. 16211157 PMC2626328

[12] Van Leeuwen MP , Slot DE , Van der Weijden GA . The effect of an essential‐oils mouthrinse as compared to a vehicle solution on plaque and gingival inflammation: a systematic review and meta‐analysis. Int J Dent Hyg. 2014;12:160‐167. 24720368 10.1111/idh.12069

[13] Van der Weijden FA , Van der Sluijs E , Ciancio SG , Slot DE . Can chemical mouthwash agents achieve plaque/gingivitis control? Dent Clin N Am. 2015;59:799‐829. 26427569 10.1016/j.cden.2015.06.002

[14] Stoeken JE , Paraskevas S , van der Weijden GA . The long‐term effect of a mouthrinse containing essential oils on dental plaque and gingivitis: a systematic review. J Periodontol. 2007;78:1218‐1228. 17608576 10.1902/jop.2007.060269

[15] Eldridge KR , Finnie SF , Stephens JA , Mauad AM , Munoz CA , Kettering JD . Efficacy of an alcohol‐free chlorhexidine mouthrinse as an antimicrobial agent. J Prosthet Dent. 1998;80(6):685‐690. 9830074 10.1016/s0022-3913(98)70056-3

[16] Charles CA , Amini P , Gallob J , Shang H , McGuire JA , Costa R . Antiplaque and antigingivitis efficacy of an alcohol‐free essential‐oil containing mouthrinse: a 2‐week clinical trial. Am J Dent. 2012;25(4):195‐198. 23082381

[17] Higgins JPT , Thomas J , Chandler J , et al. Cochrane Handbook for Systematic Reviews of Interventions version 6.4 (updated August 2023). 2022. Accessed January 21, 2024. www.training.cochrane.org.handbook

[18] Page MJ , McKenzie JE , Bossuyt PM , et al. The PRISMA 2020 statement: an updated guideline for reporting systematic reviews. BMJ. 2021;372:n71. 33782057 10.1136/bmj.n71 PMC8005924

[19] Shamseer L , Moher D , Clarke M , et al. Preferred reporting items for systematic review and meta‐analysis protocols (PRISMA‐P) 2015: elaboration and explanation. BMJ. 2015;350:g7647. 25555855 10.1136/bmj.g7647

[20] PROSPERO . International prospective register of systematic reviews. Accessed January 21, 2024. http://www.crd.york.ac.uk/PROSPERO

[21] Harrison H , Griffin SJ , Kuhn I , Usher‐Smith JA . Software tools to support title and abstract screening for systematic reviews in healthcare: an evaluation. BMC Med Res Methodol. 2020;20(1):7. 31931747 10.1186/s12874-020-0897-3 PMC6958795

[22] Ouzzani M , Hammady H , Fedorowicz Z , Elmagarmid A . Rayyan‐a web and mobile app for systematic reviews. Syst Rev. 2016;5(1):210. 27919275 10.1186/s13643-016-0384-4 PMC5139140

[23] Sterne JA , Hernan MA , Reeves BC , et al. ROBINS‐I: a tool for assessing risk of bias in non‐randomised studies of interventions. BMJ. 2016;355:i4919. 27733354 10.1136/bmj.i4919 PMC5062054

[24] Sterne JAC , Savovic J , Page MJ , et al. RoB 2: a revised tool for assessing risk of bias in randomised trials. BMJ. 2019;366:l4898. 31462531 10.1136/bmj.l4898

[25] McGuinness LA , Higgins JPT . Risk‐of‐bias VISualization (robvis): an R package and shiny web app for visualizing risk‐of‐bias assessments. Res Synth Methods. 2021;12(1):55‐61. 32336025 10.1002/jrsm.1411

[26] Sambunjak D , Nickerson JW , Poklepovic T , et al. Flossing for the management of periodontal diseases and dental caries in adults. Cochrane Database Syst Rev. 2011;7(12):CD008829. 10.1002/14651858.CD008829.pub2 22161438

[27] Egger M , Davey Smith G , Schneider M , Minder C . Bias in meta‐analysis detected by a simple, graphical test. BMJ. 1997;315(7109):629‐634. 9310563 10.1136/bmj.315.7109.629 PMC2127453

[28] Ryan R . Heterogeneity and Subgroup Analyses in Cochrane Consumers and Communication Review Group Reviews: Planning the Analysis at Protocol Stage. Cochrane Consumers and Communication Review Group; 2016. Accessed January 21, 2024. http://cccrg.cochrane.org.

[29] GRADE Wokring Group . Grading of recommendations assessment, development and evaluation working group. Accessed January 21, 2024. http://www.gradeworkinggroup.org/

[30] Guyatt GH , Oxman AD , Kunz R , et al. Incorporating considerations of resources use into grading recommendations. BMJ. 2008;336(7654):1170‐1173. 18497416 10.1136/bmj.39504.506319.80 PMC2394579

[31] Basso M , Nowakowska J , Bordini G . Valutazione clinica comparativa fra 2 collutoriagli oli essenziali con e senza alcool. Prev Assist Dent. 2011;37(2):57‐66.

[32] Marchetti E , Mummolo S , Di Mattia J , et al. Efficacy of essential oil mouthwash with and without alcohol: a 3‐day plaque accumulation model. Trials. 2011;12:262. 22171999 10.1186/1745-6215-12-262 PMC3292473

[33] Pizzo G , Compilato D , Di Liberto B , Pizzo I , Campisi G . Effects of two essential oil mouthrinses on 4‐day supragingival plaque regrowth: a randomized cross‐over study. Am J Dent. 2013;26(3):156‐160. 23986963

[34] Marchetti E , Tecco S , Caterini E , et al. Alcohol‐free essential oils containing mouthrinse efficacy on three‐day supragingival plaque regrowth: a randomized crossover clinical trial. Trials. 2017;18(1):154. 28359280 10.1186/s13063-017-1901-z PMC5374648

[35] Lynch MC , Cortelli SC , McGuire JA , et al. The effects of essential oil mouthrinses with or without alcohol on plaque and gingivitis: a randomized controlled clinical study. BMC Oral Health. 2018;18(1):6. 29321067 10.1186/s12903-017-0454-6 PMC5763666

[36] Spuldaro TR , Dos Santos R , Junior M , de Oliveira V , Fernandes G , Rosing CK . Efficacy of essential oil mouthwashes with and without alcohol on the plaque formation: a randomized, crossover, double‐blinded, clinical trial. J Evid Based Dent Pract. 2021;21(1):101527. 34051963 10.1016/j.jebdp.2021.101527

[37] Bosma ML , McGuire JA , DelSasso A , Milleman J , Milleman K . Efficacy of flossing and mouth rinsing regimens on plaque and gingivitis: a randomized clinical trial. BMC Oral Health. 2024;24(1):178. 38310236 10.1186/s12903-024-03924-4 PMC10837857

[38] Tanner AC , Kent R Jr , Van Dyke T , Sonis ST , Murray LA . Clinical and other risk indicators for early periodontitis in adults. J Periodontol. 2005;76(4):573‐581. 15857098 10.1902/jop.2005.76.4.573 PMC1224718

[39] Turesky S , Gilmore ND , Glickman I . Reduced plaque formation by the chloromethyl analogue of victamine C. J Periodontol. 1970;41(1):41‐43. 5264376 10.1902/jop.1970.41.41.41

[40] Quigley GA , Hein JW . Comparative cleansing efficiency of manual and power brushing. J Am Dent Assoc. 1962;65:26‐29. 14489483 10.14219/jada.archive.1962.0184

[41] Moher D , Liberati A , Tetzlaff J , Altman DG , Group P . Preferred reporting items for systematic reviews and meta‐analyses: the PRISMA statement. PLoS Med. 2009;6(7):e1000097. 19621072 10.1371/journal.pmed.1000097 PMC2707599

[42] Grunevald M , Cantarelli R , Oballe HJR , et al. Antimicrobial potential of essential oils mouthrinses with and without alcohol: a randomized clinical trial. Scopus. 2021;20:1‐9.

[43] American Dental Association (ADA). Acceptance Program Guidelines . Chemotherapeutic Products for Control of Gingivitis. 2016. Accessed January 18, 2024. https://www.ada.org

[44] Newcombe RG , Addy M , McKeown S . Residual effect of chlorhexidine gluconate in 4‐day plaque regrowth crossover trials, and its implications for study design. J Periodontal Res. 1995;30(5):319‐324. 7494173 10.1111/j.1600-0765.1995.tb01282.x

[45] DePaola LG , Spolarich AE . Safety and efficacy of antimicrobial Mouthrinses in clinical practice. J Dent Hyg. 2007;81(5):13‐25.

[46] Statista Research Department . Top mouthwash/dental rinse brands in the U.S. 2018. Accessed January 18, 2024. https://www.statista.com/statistics/195543/sales‐of‐leading‐us‐mouthwash‐brands‐in‐2012‐and‐2013/

[47] Botelho MA , dos Santos RA , Martins JG , et al. Comparative effect of an essential oil mouthrinse on plaque, gingivitis and salivary Streptococcus mutans levels: a double blind randomized study. Phytother Res. 2009;23(9):1214‐1219. 19370543 10.1002/ptr.2489

[48] Botelho MA , Bezerra Filho JG , Correa LL , et al. Effect of a novel essential oil mouthrinse without alcohol on gingivitis: a double‐blinded randomized controlled trial. J Appl Oral Sci. 2007;15(3):175‐180. 19089126 10.1590/S1678-77572007000300005 PMC4327463

[49] Girao VC , Nunes‐Pinheiro DC , Morais SM , Sequeira JL , Gioso MA . A clinical trial of the effect of a mouth‐rinse prepared with Lippia sidoides Cham essential oil in dogs with mild gingival disease. Prev Vet Med. 2003;59(1–2):95‐102. 12719020 10.1016/s0167-5877(03)00051-5

[50] Haq MW , Batool M , Ahsan SH , Qureshi NR . Alcohol use in mouthwash and possible oral health concerns. J Pak Med Assoc. 2009;59(3):186‐190. 19288954

[51] Eley BM . Antibacterial agents in the control of supragingival plaque – a review. Br Dent J. 1999;186(6):286‐296. 10230103 10.1038/sj.bdj.4800090

[52] Wynder EL , Kabat G , Rosenberg S , Levenstein M . Oral cancer and mouthwash use. J Natl Cancer Inst. 1983;70(2):255‐260. 6571934

[53] Elmore JG , Horwitz RI . Oral cancer and mouthwash use: evaluation of the epidemiologic evidence. Otolaryngol Head Neck Surg. 1995;113(3):253‐261. 7675486 10.1016/S0194-5998(95)70114-1

[54] Ahrens W , Pohlabeln H , Foraita R , et al. Oral health, dental care and mouthwash associated with upper aerodigestive tract cancer risk in Europe: the ARCAGE study. Oral Oncol. 2014;50(6):616‐625. 24680035 10.1016/j.oraloncology.2014.03.001

[55] La Vecchia C . Mouthwash and oral cancer risk: an update. Oral Oncol. 2009;45(3):198‐200. 18952488 10.1016/j.oraloncology.2008.08.012

[56] Gandini S , Negri E , Boffetta P , La Vecchia C , Boyle P . Mouthwash and oral cancer risk quantitative meta‐analysis of epidemiologic studies. Ann Agric Environ Med. 2012;19(2):173‐180. 22742785

[57] Ustrell‐Borras M , Traboulsi‐Garet B , Gay‐Escoda C . Alcohol‐based mouthwash as a risk factor of oral cancer: a systematic review. Med Oral Patol Oral Cir Bucal. 2020;25(1):e1‐e12. 31655832 10.4317/medoral.23085 PMC6982979

[58] Kumar M , Nanavati R , Modi TG , Dobariya C . Oral cancer: etiology and risk factors: a review. J Cancer Res Ther. 2016;12(2):458‐463. 27461593 10.4103/0973-1482.186696

[59] Dayyani F , Etzel CJ , Liu M , Ho CH , Lippman SM , Tsao AS . Meta‐analysis of the impact of human papillomavirus (HPV) on cancer risk and overall survival in head and neck squamous cell carcinomas (HNSCC). Head Neck Oncol. 2010;2:15. 20587061 10.1186/1758-3284-2-15 PMC2908081

[60] Tanaka TI , Alawi F . Human papillomavirus and oropharyngeal cancer. Dent Clin N Am. 2018;62(1):111‐120. 29126488 10.1016/j.cden.2017.08.008

[61] Leal JP , da Silva JD , Leal RFM , Oliveira‐Junior CDC , Prado VLG , Vale GC . Effect of mouthwashes on solubility and sorption of restorative composites. Int J Dentistry. 2017;2017:5865691. 10.1155/2017/5865691 PMC5480039 28684960

[62] Prado V , Santos K , Fontenele R , Soares J , Vale G . Effect of over the counter mouthwashes with and without alcohol on sorption and solubility of bulk fill resins. J Clin Exp Dent. 2020;12(12):e1150‐e1156. 33282136 10.4317/jced.57234 PMC7700788

[63] Almeida GS , Poskus LT , Guimaraes JG , da Silva EM . The effect of mouthrinses on salivary sorption, solubility and surface degradation of a nanofilled and a hybrid resin composite. Oper Dent. 2010;35(1):105‐111. 20166417 10.2341/09-080-L

[64] Pelino JEP , Passero A , Martin AA , Charles CA . In vitro effects of alcohol‐containing mouthwashes on human enamel and restorative materials. Braz Oral Res. 2018;32:e25. 29561951 10.1590/1807-3107bor-2018.vol32.0025

[65] Gunsolley JC . A meta‐analysis of six‐month studies of antiplaque and antigingivitis agents. J Am Dent Assoc. 2006;137(12):1649‐1657. 17138709 10.14219/jada.archive.2006.0110

[66] Gunsolley JC . Clinical efficacy of antimicrobial mouthrinses. J Dent. 2010;38(Suppl 1):S6‐S10. 20621242 10.1016/S0300-5712(10)70004-X

[67] Araujo MWB , Charles CA , Weinstein RB , et al. Meta‐analysis of the effect of an essential oil‐containing mouthrinse on gingivitis and plaque. J Am Dent Assoc. 2015;146(8):610‐622. 26227646 10.1016/j.adaj.2015.02.011

[68] Escribano M , Figuero E , Martin C , et al. Efficacy of adjunctive anti‐plaque chemical agents: a systematic review and network meta‐analyses of the Turesky modification of the Quigley and Hein plaque index. J Clin Periodontol. 2016;43(12):1059‐1073. 27531174 10.1111/jcpe.12616

[69] Serrano J , Escribano M , Roldan S , Martin C , Herrera D . Efficacy of adjunctive anti‐plaque chemical agents in managing gingivitis: a systematic review and meta‐analysis. J Clin Periodontol. 2015;42(Suppl 16):S106‐S138. 25495592 10.1111/jcpe.12331

[70] Figuero E , Herrera D , Tobias A , et al. Efficacy of adjunctive anti‐plaque chemical agents in managing gingivitis: a systematic review and network meta‐analyses. J Clin Periodontol. 2019;46(7):723‐739. 31058336 10.1111/jcpe.13127

[71] Van der Weijden FA , Slot DE . Efficacy of homecare regimens for mechanical plaque removal in managing gingivitis a meta review. J Clin Periodontol. 2015;42(Suppl 16):S77‐S91. 25597787 10.1111/jcpe.12359

[72] Haas AN , Wagner TP , Muniz F , Fiorini T , Cavagni J , Celeste RK . Essential oils‐containing mouthwashes for gingivitis and plaque: meta‐analyses and meta‐regression. J Dent. 2016;55:7‐15. 27628316 10.1016/j.jdent.2016.09.001

[73] Quintas V , Prada‐Lopez I , Carreira MJ , Suarez‐Quintanilla D , Balsa‐Castro C , Tomas I . In situ antibacterial activity of essential oils with and without alcohol on Oral biofilm: a randomized clinical trial. Front Microbiol. 2017;8:2162. 29218030 10.3389/fmicb.2017.02162 PMC5703870

[74] Ainamo J , Bay I . Problems and proposals for recording gingivitis and plaque. Int Dent J. 1975;25(4):229‐235. 1058834

[75] Lobene RR , Weatherford T , Ross NM , Lamm RA , Menaker L . A modified gingival index for use in clinical trials. Clin Prev Dent. 1986;8(1):3‐6. 3485495

[76] BMJ best practice. What is GRADE? 2024. Accessed January 21, 2024. https://bestpractice.bmj.com/info/toolkit/learn‐ebm/what‐is‐grade

