# Supplementary material for: Essential oils mouthwash with or without alcohol in relation to effect on parameters of plaque and gingivitis: A systematic review and meta‐analysis
Source: Int J Dent Hyg. 2024 Aug 12;23(1):186–202. doi: 10.1111/idh.12843 (PMC11717972; doi:10.1111/idh.12843)
Supplement: Supplementary file 1 — Appendix S1–S10. [file IDH-23-186-s001.pdf]

Essential oils mouthwash with or without alcohol in relation to effect on parameters of plaque and gingivitis.
-A systematic review and meta-analysis-

Online Supporting Information

B.W.M. van Swaaij*/**

G.A. Van der Weijden*

R.J. Smith*

M.F. Timmerman ***

D.E. Slot*

Author affiliations:

* Department of Periodontology. Academic Centre for Dentistry Amsterdam (ACTA), University of Amsterdam and Vrije Universiteit Amsterdam, The Netherlands.Department of Dental Hygiene.

** Department of Dental Hygiene. Hogeschool Arnhem Nijmegen, University of Applied Sciences, Nijmegen, The Netherlands

*** Department of Implantology and Periodontology. Radboud University Medical Center (Radboudumc), The Netherlands

Online Supporting Information Legends

ONLINE Appendix S1
Methodological quality and potential risk of bias scores of the individual included studies.

ONLINE Appendix S2
Mean (SD) scores for the different intervention groups, including various indices and their modifications.

ONLINE Appendix S3
Forest plot: for the non-brushing design; plaque scores analyzed by the Turesky modification of the Quigley-Hein plaque index

ONLINE Appendix S4
Forest plot: for the brushing design; plaque scores analyzed by the Turesky modification of the Quigley-Hein plaque index

ONLINE Appendix S5
Forest plot: for the brushing design; bleeding scores analyzed by Ainamo and Bay (1975)

ONLINE Appendix S6
Forest plot: for the brushing design; gingival scores analyzed by Lobene (1986).

ONLINE Appendix S7
Forest plot: taste perception analyzed by visual analog scale.

ONLINE Appendix S8
Forest plot: taste remain analyzed by visual analog scale.

ONLINE Appendix S9
Forest plot: taste alteration analyzed by visual analog scale.

ONLINE Appendix S10
PRISMA 2009 Checklist as proposed by Page (2020)

Appendix S1a

Methodological quality and potential risk of bias scores for the individual included studies.

Traffic light: displaying the risk of bias judgement for each domain for each study.

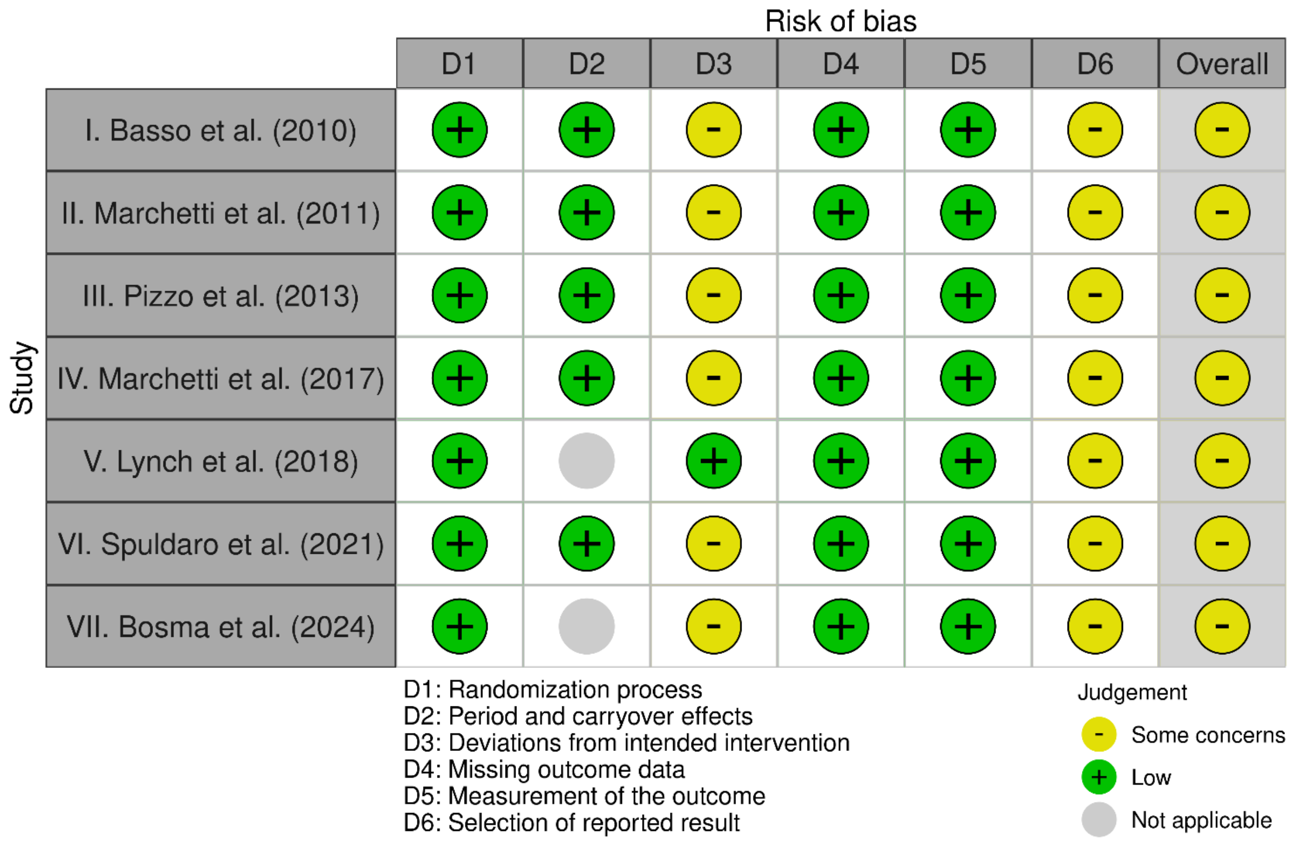

Tool used: https://www.riskofbias.info/welcome/robvis-visualization-tool

Appendix S1b

Methodological quality and potential risk of bias scores for the individual included studies.

Summary plot: weighted bar plots of the distribution of risk-of-bias judgements within each bias domain.

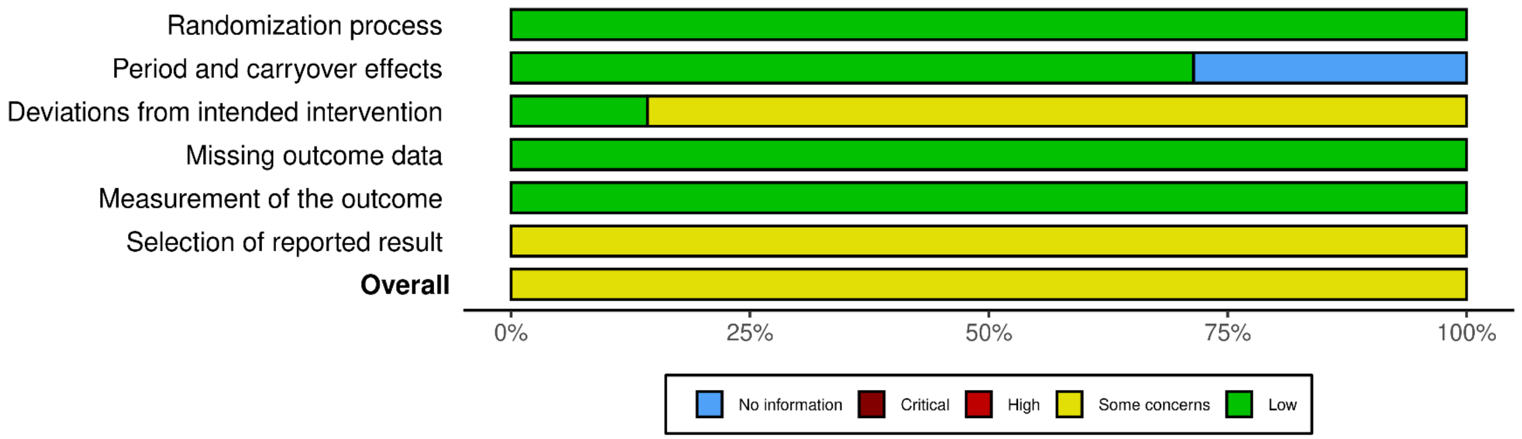

Tool used: https://www.riskofbias.info/welcome/robvis-visualization-tool

Appendix S2

ONLINE appendix 2a					
**# PI**	**Index %**	**Group**	**Baseline**	**End**	**Difference**
Marchetti et al. (2011) NB	Turesky modification of the Quigley-Hein Plaque Index (1970)	MW EOalc-	‡	2.46 (0.42)	NA
		MW EOalc+	‡	2.18 (0.39)	NA
Marchetti et al. (2017) NB	Turesky modification of the Quigley-Hein Plaque Index, modified by Lobene et al. (1982)	MW EOalc-	‡	1.72 (0.36)	NA
		MW EOalc+	‡	1.65 (0.35)	NA
Pizzo et al. (2013) NB	Turesky modification of the Quigley-Hein Plaque Index (1970)	MW EOalc-	‡	3.17 (0.38)	NA
		MW EOalc+	‡	1.86 (0.37)	NA
Bosma et al. (2024) B	Turesky Plaque Index (?)	MW EOalc-	3.00 (0.364)	2.14 (0.324◊)	-0.86◊ (??)
		MW EOalc+	2.98 (0.468)	1.80 (0.325◊)	-1.18◊ (??)
Lynch et al. (2018) B	Turesky modification of the Quigley-Hein Plaque Index (1970)	MW EOalc-	2.99 (0.23) ♦	1.81 (0.18) ♦	-1.17 (0.29) ♦
		MW EOalc+	3.02 (0.27) ♦	1.80 (0.16) ♦	-1.22 (0.29) ♦
Basso et al. (2010) B	FMPS Ainamo & Bay (1975)	MW EOalc-	21.43 (22.20) ♦	15.00 (13.84) ♦	-6.43 (14.40) ♦
		MW EOalc+	23.04 (23.87) ♦	16.93 (14.36) ♦	-6.11 (12.65) ♦
Spuldaro et al. (2021) NB	Plaque Free Zone Weidlich et al. (2001)	MW EOalc-	‡	0%, 49%, 51%	NA
		ME EOalc+	‡	1%, 61%, 38%	NA

Mean (SD) scores for the different intervention groups with various indices and their modifications. Within-group analyses are presented. S2a: Plaque Index (PI), S2b: Bleeding Index (BI), S2c: Gingival Index (GI) S2d: Taste Perception (TP), S2e: Taste Remaining (TR), S2f: Taste Alteration (TA).

ONLINE appendix 2b					
**# BI**	**Index %**	**Group**	**Baseline**	**End**	**Difference**
Lynch et al. (2018) B	Ainamo and Bay (1975)	MW EOalc-	0.62 (0.16) ♦	0.18 (0.05) ♦	-0.44 (0.16) ♦
		MW EOalc+	0.63 (0.17) ♦	0.19 (0.07) ♦	-0.44 (0.16) ♦
Bosma et al. (2024) B	Whole mouth Bleeding on probing (BOP)	MW EOalc-	0.144 (0.1840)	0.045 (0.058◊)	-0.099◊ (??)
		MW EOalc+	0.099 (0.1152)	0.034 (0.058◊)	-0.065◊ (??)
Basso et al. (2010) B	FMBS % Ainamo and Bay (1975)	MW EOalc-	12.82 (19.62) ♦	8.05 (8.28) ♦	-4.77 (17.6) ♦
		MW EOalc+	13.64 (14.62) ♦	8.11 (9.34) ♦	-5.54 (8.80) ♦
	FMBS Ainamo and Bay (1975) / 100	MW EOalc-	0.13 (0.20) ♦	0,08 (0.08) ♦	-0.05 (0.18) ♦
		MW EOalc+	0.14 (0.15) ♦	0,08 (0.09) ♦	-0.06 (0.09) ♦

ONLINE appendix 2c					
**# GI**	**Index %**	**Group**	**Baseline**	**End**	**Difference**
Lynch et al. (2018) B	Modified Gingival Index – Lobene (1986)	MW EOalc-	2.45 (0.15) ♦	1.61(0.17) ♦	-0.84 (0.22) ♦
		MW EOalc+	2.47 (0.15) ♦	1.58 (0.14) ♦	-0.88 (0.20) ♦
Bosma et al. (2024) B	Modified Gingival Index	MW EOalc-	2.65 (0.288)	1.43 (0.389◊)	-1.22◊ (??)
		MW EOalc+	2.56 (0.320)	1.37 (0.389◊)	-1.19◊ (??)

ONLINE appendix 2d					
**# TP**	**Index %**	**Group**	**Baseline**	**End**	**Difference**
Marchetti et al. (2011)	Visual analog scale (VAS)	MW EOalc-	‡	6.6 (2.27)	NA
		MW EOalc+	‡	5.17 (2.73)	NA
Marchetti et al. (2017)	Visual analog scale (VAS)	MW EOalc-	‡	7.5 (1.43)	NA
		MW EOalc+	‡	5.65 (2.70)	NA

ONLINE appendix 2e					
**# TR**	**Index %**	**Group**	**Baseline**	**End**	**Difference**
Marchetti et al. (2011)	Visual analog scale (VAS)	MW EOalc-	‡	5.5 (2.36)	NA
		MW EOalc+	‡	5.97 (1.71)	NA
Marchetti et al. (2017)	Visual analog scale (VAS)	MW EOalc-	‡	5.10 (2.25)	NA
		MW EOalc+	‡	4.95 (2.65)	NA

ONLINE appendix 2f					
**# TA**	**Index %**	**Group**	**Baseline**	**End**	**Difference**
Marchetti et al. (2011)	Visual analog scale (VAS)	MW EOalc-	‡	2.97 (3.27)	NA
		MW EOalc+	‡	2.3 (3.02)	NA
Marchetti et al. (2017)	Visual analog scale (VAS)	MW EOalc-	‡	5.20 (1.96)	NA
		MW EOalc+	‡	4.70 (1.90)	NA

PI: Plaque Index, BI: Bleeding Index, GI: Gingival Index, TP: Taste Perception, TR: Taste Remaining, Ta: Taste Alteration, MW: Mouthwash, Alc+: With Alcohol, Alc-: Without Alcohol, B: Brushing study, NB: Non Brushing Study

? Unknown/not provided
₪ Insufficient data presentation
◊ Calculated by the authors of this review based on the presented data in the selected paper
♦ Provided by the original author
‡ Not measured
NA Not applicable

Appendix S3

3a. For the non-brushing study design, plaque scores evaluated analyzed by the Turesky modification of the Quigley-Hein plaque index (1970). Forest plots of the performed meta-analysis using a fixed model evaluating EOalc- MW compared to EOalc+ MW on end scores.

	EO without alc			EO with alc				Mean Difference
**Study or Subgroup**	**Mean**	**SD**	**Total**	**Mean**	**SD**	**Total**	**Weight**	**IV, Fixed, 95% CI**
Marchetti 2011	2.46	0.42	30	2.18	0.39	30	42.0%	0.28 [0.07, 0.49]
Marchetti 2017	1.72	0.36	21	1.65	0.35	21	38.3%	0.07 [-0.14, 0.28]
Pizzo 2013	3.17	0.38	12	1.86	0.37	12	19.6%	1.31 [1.01, 1.61]
**Total (95% CI)**			**63**			**63**	**100.0%**	**0.40 [0.27, 0.53]**

Heterogeneity: Chi² = 45.71, df = 2 (P < 0.00001); I² = 96%
Test for overall effect: Z = 5.92 (P < 0.00001)

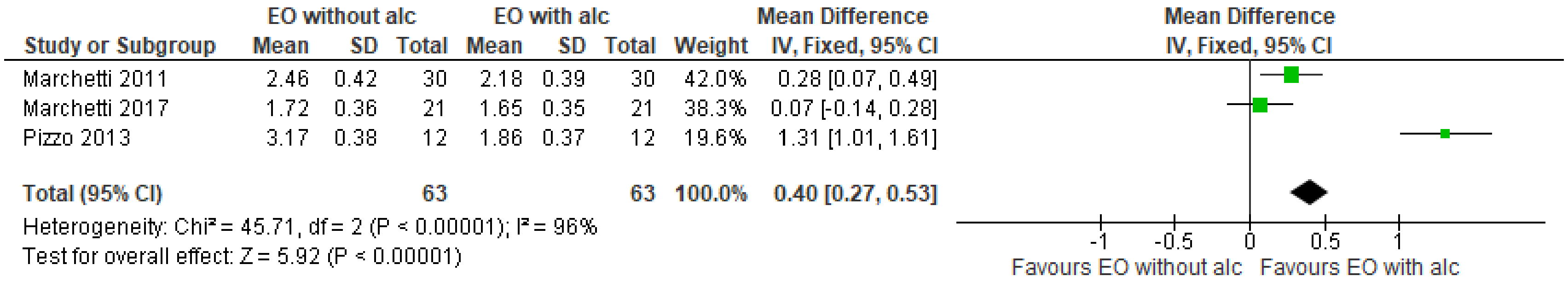

3b. Fixed model of the performed meta-analysis for evaluating the sub analysis regarding the EOalc- MW groups using Curacept® Daycare, compared EOalc+ MW

	EO without Alc			EO with Alc				Mean Difference
**Study or Subgroup**	**Mean**	**SD**	**Total**	**Mean**	**SD**	**Total**	**Weight**	**IV, Fixed, 95% CI**
**5.1.2 Curacept**								
Marchetti 2011	2.46	0.42	30	2.18	0.39	30	68.2%	0.28 [0.07, 0.49]
Pizzo 2013	3.17	0.38	12	1.86	0.37	12	31.8%	1.31 [1.01, 1.61]
**Subtotal (95% CI)**			**42**			**42**	**100.0%**	**0.61 [0.44, 0.78]**
Heterogeneity: Chi² = 30.85, df = 1 (P < 0.00001); I² = 97%								
Test for overall effect: Z = 7.04 (P < 0.00001)								
**Total (95% CI)**			**42**			**42**	**100.0%**	**0.61 [0.44, 0.78]**
Heterogeneity: Chi² = 30.85, df = 1 (P < 0.00001); I² = 97%								
Test for overall effect: Z = 7.04 (P < 0.00001)								
Test for subgroup differences: Not applicable								

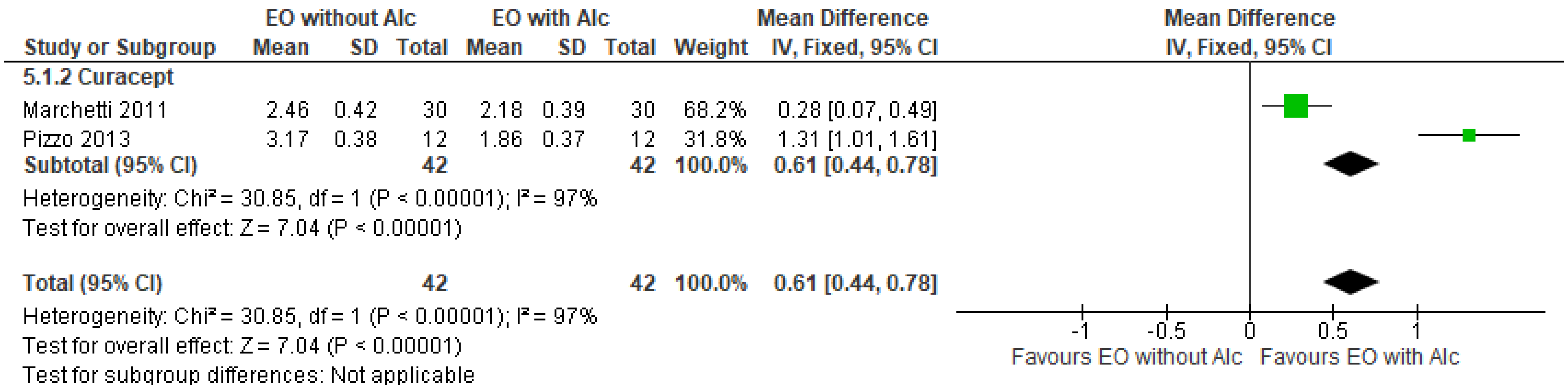

Appendix S4

For the brushing design, plaque scores evaluated analyzed by the Turesky modification of the Quigley-Hein plaque index (1970). Forest plots of the performed meta-analysis using a fixed model evaluating EOalc- MW compared to EOalc+ MW.

4a. Baseline

	Alc-			Alc+				Mean Difference
**Study or Subgroup**	**Mean**	**SD**	**Total**	**Mean**	**SD**	**Total**	**Weight**	**IV, Fixed, 95% CI**
Bosma 2024	3	0.364	52	2.98	0.468	51	13.1%	0.02 [-0.14, 0.18]
Lynch 2018	2.991	0.23	121	3.017	0.27	122	86.9%	-0.03 [-0.09, 0.04]
**Total (95% CI)**			**173**			**173**	**100.0%**	**-0.02 [-0.08, 0.04]**

Heterogeneity: Chi² = 0.27, df = 1 (P = 0.60); I² = 0%

Test for overall effect: Z = 0.67 (P = 0.51)

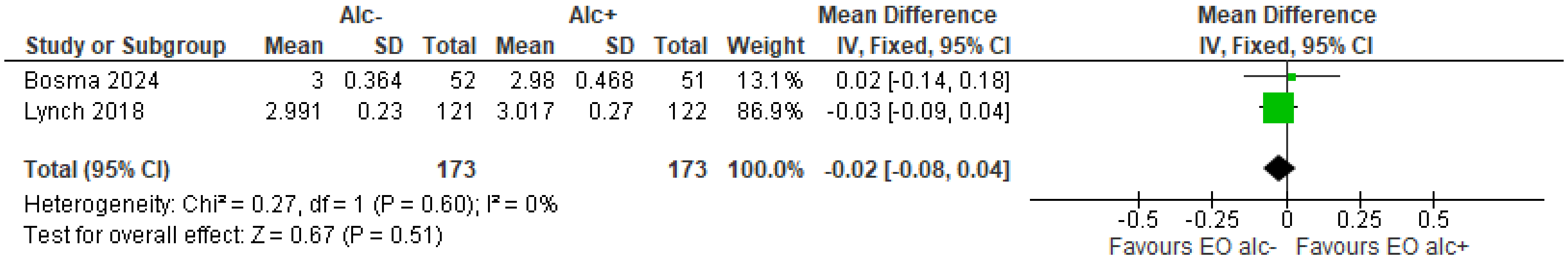

4b. End

	Alc-			Alc+				Mean Difference
**Study or Subgroup**	**Mean**	**SD**	**Total**	**Mean**	**SD**	**Total**	**Weight**	**IV, Fixed, 95% CI**
Bosma 2024	2.14	0.324	52	1.8	0.325	50	11.2%	0.34 [0.21, 0.47]
Lynch 2018	1.814	0.1847	116	1.795	0.1608	114	88.8%	0.02 [-0.03, 0.06]
**Total (95% CI)**			**168**			**164**	**100.0%**	**0.05 [0.01, 0.10]**

Heterogeneity: Chi² = 22.15, df = 1 (P < 0.00001); I² = 95%

Test for overall effect: Z = 2.55 (P = 0.01)

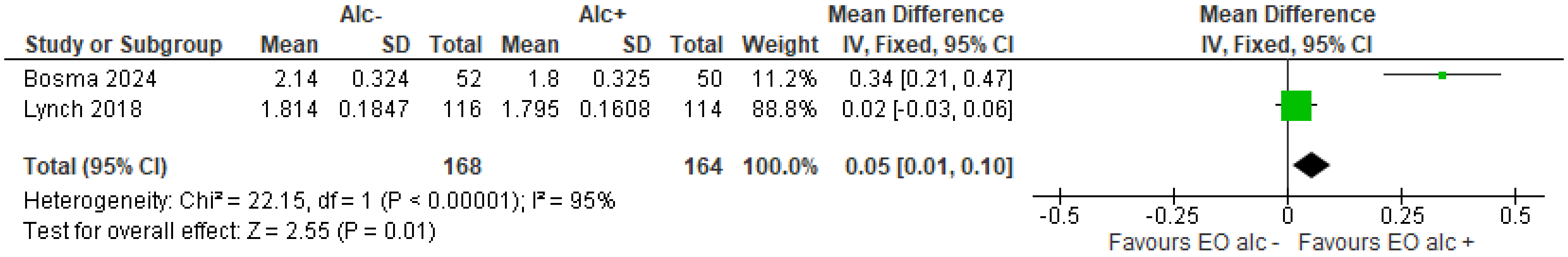

Appendix S5

For the brushing design, bleeding scores analyzed by Ainamo and Bay (1975). Forest plots of the performed meta-analysis evaluating EOalc- MW compared to EOalc+ MW.

a. Baseline

	EO without alc			EO with alc				Mean Difference	Mean Difference
**Study or Subgroup**	**Mean**	**SD**	**Total**	**Mean**	**SD**	**Total**	**Weight**	**IV, Fixed, 95% CI**	**IV, Fixed, 95% CI**
Basso 2010	0.128	0.196	28	0.136	0.146	28	18.4%	-0.01 [-0.10, 0.08]	
Lynch 2018	0.623	0.1649	116	0.627	0.1674	114	81.6%	-0.00 [-0.05, 0.04]	
**Total (95% CI)**			**144**			**142**	**100.0%**	**-0.00 [-0.04, 0.03]**	

Heterogeneity: Chi² = 0.01, df = 1 (P = 0.94); I² = 0%
Test for overall effect: Z = 0.24 (P = 0.81)

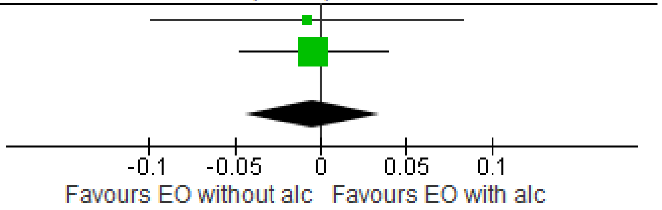

b. End

	EO without alc			EO with alc				Mean Difference	Mean Difference
**Study or Subgroup**	**Mean**	**SD**	**Total**	**Mean**	**SD**	**Total**	**Weight**	**IV, Fixed, 95% CI**	**IV, Fixed, 95% CI**
Basso 2010	0.081	0.083	28	0.081	0.093	28	9.9%	0.00 [-0.05, 0.05]	
Lynch 2018	0.181	0.0496	116	0.187	0.0672	114	90.1%	-0.01 [-0.02, 0.01]	
**Total (95% CI)**			**144**			**142**	**100.0%**	**-0.01 [-0.02, 0.01]**	

Heterogeneity: Chi² = 0.06, df = 1 (P = 0.81); I² = 0%
Test for overall effect: Z = 0.73 (P = 0.47)

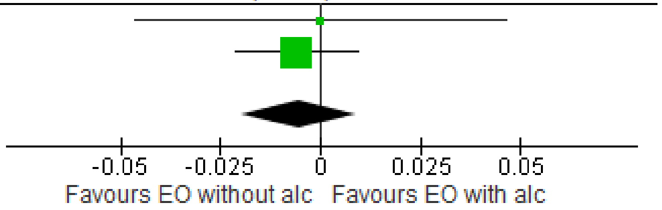

c. Difference

Study or Subgroup	EO without alc			EO with alc			Weight	Mean Difference
	Mean	SD	Total	Mean	SD	Total		IV, Fixed, 95% CI
Basso 2010	-0.048	0.176	28	-0.055	0.088	28	23.6%	0.01 [-0.07, 0.08]
Lynch 2018	-0.442	0.1581	116	-0.44	0.1552	114	76.4%	-0.00 [-0.04, 0.04]
**Total (95% CI)**			**144**			**142**	**100.0%**	**0.00 [-0.04, 0.04]**

Heterogeneity: $Chi^2 = 0.04, df = 1 (P = 0.83); I^2 = 0\%$
Test for overall effect: $Z = 0.01 (P = 0.99)$

Mean Difference
IV, Fixed, 95% CI

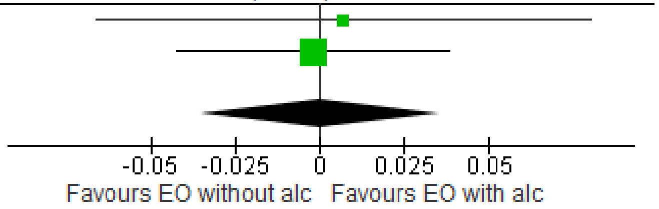

Appendix S6

For the brushing design, gingival scores analyzed by Modified Gingival Index by Lobene (1986). Forest plots of the performed meta-analysis evaluating EOalc- MW compared to EOalc+ MW.

a. Baseline

	Alc-			Alc+				Mean Difference
**Study or Subgroup**	**Mean**	**SD**	**Total**	**Mean**	**SD**	**Total**	**Weight**	**IV, Fixed, 95% CI**
Bosma 2024	2.65	0.288	52	2.56	0.32	51	9.3%	0.09 [-0.03, 0.21]
Lynch 2018	2.452	0.15	121	2.463	0.15	122	90.7%	-0.01 [-0.05, 0.03]
**Total (95% CI)**			**173**			**173**	**100.0%**	**-0.00 [-0.04, 0.03]**

Heterogeneity: Chi² = 2.57, df = 1 (P = 0.11); I² = 61%
Test for overall effect: Z = 0.09 (P = 0.93)

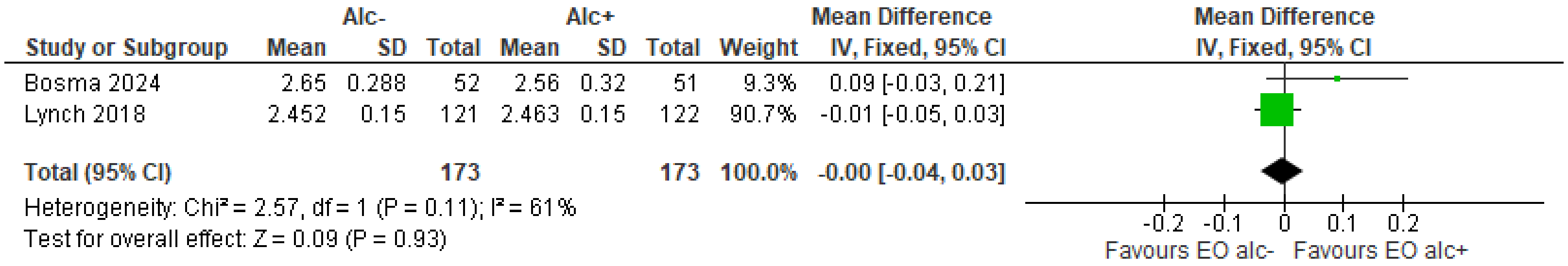

b. End

	Alc-			Alc+				Mean Difference
**Study or Subgroup**	**Mean**	**SD**	**Total**	**Mean**	**SD**	**Total**	**Weight**	**IV, Fixed, 95% CI**
Bosma 2024	1.43	0.389	52	1.37	0.389	50	6.3%	0.06 [-0.09, 0.21]
Lynch 2018	1.614	0.17	121	1.581	0.14	122	93.7%	0.03 [-0.01, 0.07]
**Total (95% CI)**			**173**			**172**	**100.0%**	**0.03 [-0.00, 0.07]**

Heterogeneity: Chi² = 0.12, df = 1 (P = 0.73); I² = 0%
Test for overall effect: Z = 1.79 (P = 0.07)

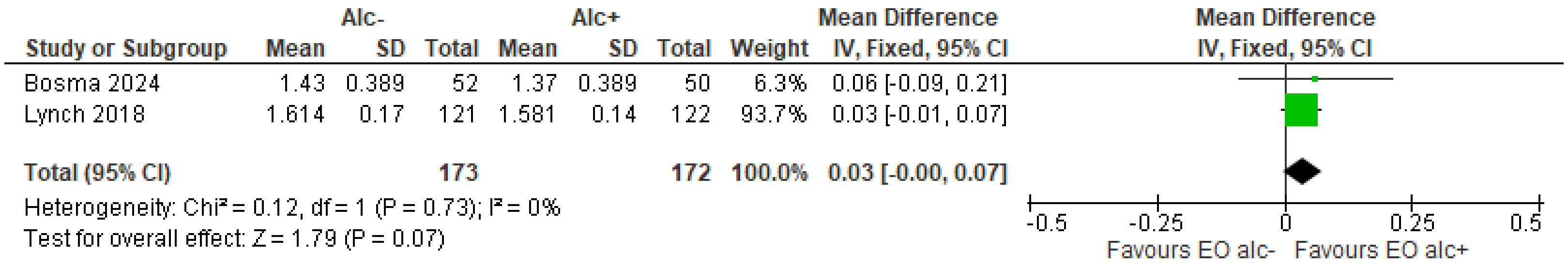

Appendix S7

Taste perception evaluated as the <u>negative taste</u> of the MW product itself. Forest plots of the performed meta-analysis using a fixed model analyzing visual analog scale of EOalc- MW compared to EOalc+ MW.

	EO without alc			EO with alc				Mean Difference
**Study or Subgroup**	**Mean**	**SD**	**Total**	**Mean**	**SD**	**Total**	**Weight**	**IV, Fixed, 95% CI**
Marchetti 2011	6.6	2.27	30	5.17	2.73	30	51.4%	1.43 [0.16, 2.70]
Marchetti 2017	7.5	1.43	21	5.65	2.7	21	48.6%	1.85 [0.54, 3.16]
**Total (95% CI)**			**51**			**51**	**100.0%**	**1.63 [0.72, 2.55]**

Heterogeneity: Chi² = 0.20, df = 1 (P = 0.65); I² = 0%
Test for overall effect: Z = 3.52 (P = 0.0004)

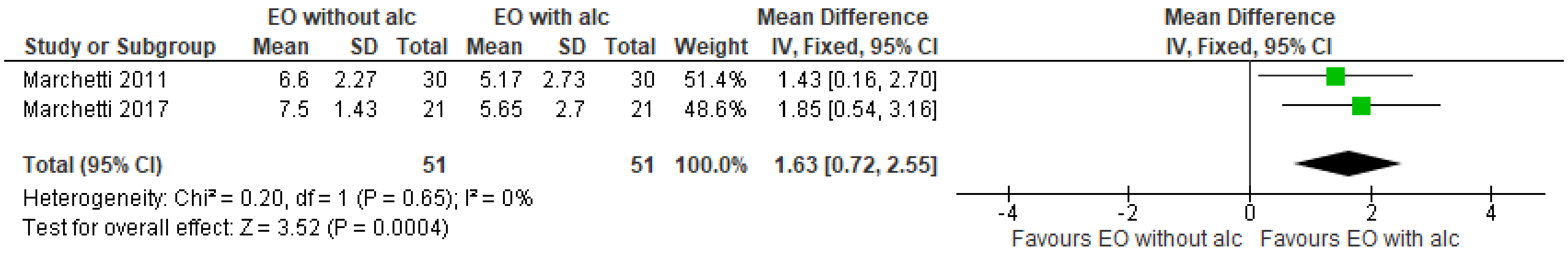

Appendix S8

Taste remain evaluated as how long the taste remained in the mouth after rinsing. Forest plots of the performed meta-analysis using a fixed model analyzing visual analog scale of EOalc- MW compared to EOalc+ MW.

	EO without alc			EO with alc				Mean Difference
**Study or Subgroup**	**Mean**	**SD**	**Total**	**Mean**	**SD**	**Total**	**Weight**	**IV, Fixed, 95% CI**
Marchetti 2011	5.5	2.36	30	5.97	1.71	30	67.0%	-0.47 [-1.51, 0.57]
Marchetti 2017	5.1	2.25	21	4.95	2.65	21	33.0%	0.15 [-1.34, 1.64]
**Total (95% CI)**			**51**			**51**	**100.0%**	**-0.27 [-1.12, 0.59]**

Heterogeneity: Chi² = 0.45, df = 1 (P = 0.50); I² = 0%
Test for overall effect: Z = 0.61 (P = 0.54)

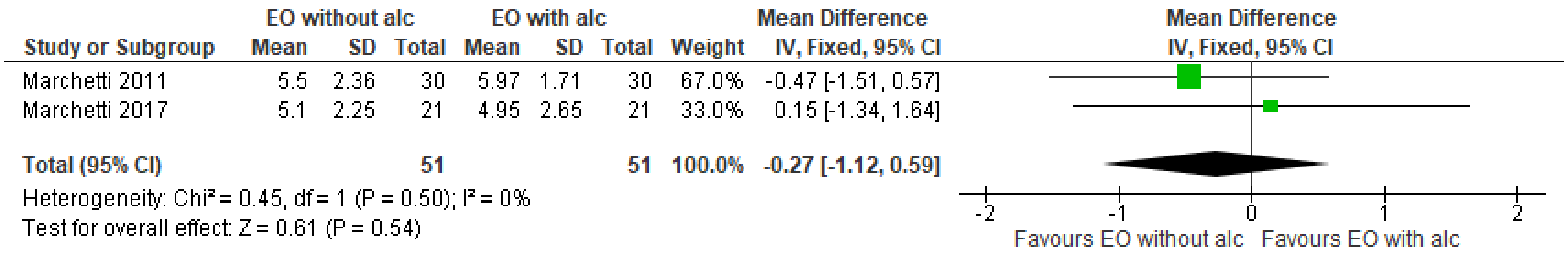

Appendix S9

Taste alteration evaluated as affecting more negative the taste on food and drinks. Forest plots of the performed meta-analysis using a fixed model analyzing visual analog scale of EOalc- MW compared to EOalc+ MW.

	EO without alc			EO with alc				Mean Difference
**Study or Subgroup**	**Mean**	**SD**	**Total**	**Mean**	**SD**	**Total**	**Weight**	**IV, Fixed, 95% CI**
Marchetti 2011	2.97	3.27	30	2.3	3.02	30	34.9%	0.67 [-0.92, 2.26]
Marchetti 2017	5.2	1.96	21	4.7	1.9	21	65.1%	0.50 [-0.67, 1.67]
**Total (95% CI)**			**51**			**51**	**100.0%**	**0.56 [-0.38, 1.50]**

Heterogeneity: Chi² = 0.03, df = 1 (P = 0.87); I² = 0%
Test for overall effect: Z = 1.16 (P = 0.24)

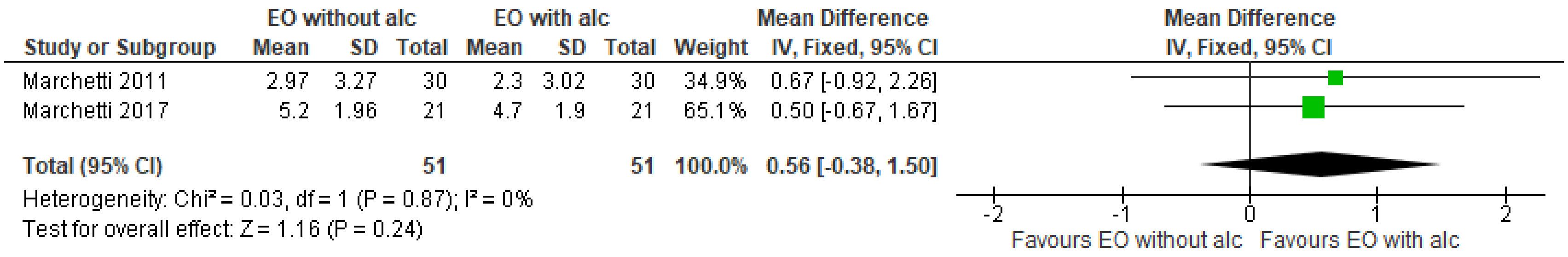

Appendix S10

PRISMA 2020 Checklist as proposed by Page (2020)

Authors: B.W.M. van Swaaij, G.A. Van der Weijden, R.J. Smith, M.F. Timmerman, D.E. Slot

Title: Essential oils mouthwash with or without alcohol in relation to effect on parameters of plaque and gingivitis. A systematic review and meta-analysis

Submitted to: International Journal of Dental Hygiene

Section and Topic	Item #	Checklist item	Location where item is reported
**TITLE**			
Title	1	Identify the report as a systematic review.	1
**ABSTRACT**			
Abstract	2	See the PRISMA 2020 for Abstracts checklist.	4
**INTRODUCTION**			
Rationale	3	Describe the rationale for the review in the context of existing knowledge.	7
Objectives	4	Provide an explicit statement of the objective(s) or question(s) the review addresses.	7
**METHODS**			
Eligibility criteria	5	Specify the inclusion and exclusion criteria for the review and how studies were grouped for the syntheses.	8
Information sources	6	Specify all databases, registers, websites, organisations, reference lists and other sources searched or consulted to identify studies. Specify the date when each source was last searched or consulted.	8
Search strategy	7	Present the full search strategies for all databases, registers and websites, including any filters and limits used.	8
Selection process	8	Specify the methods used to decide whether a study met the inclusion criteria of the review, including how many reviewers screened each record and each report retrieved, whether they worked independently, and if applicable, details of automation tools used in the process.	9
Data collection process	9	Specify the methods used to collect data from reports, including how many reviewers collected data from each report, whether they worked independently, any processes for obtaining or confirming data from study investigators, and if applicable, details of automation tools used in the process.	10
Data items	10a	List and define all outcomes for which data were sought. Specify whether all results that were compatible with each outcome domain in each study were sought (e.g. for all measures, time points, analyses), and if not, the methods used to decide which results to collect.	10
	10b	List and define all other variables for which data were sought (e.g. participant and intervention characteristics, funding sources). Describe any assumptions made about any missing or unclear information.	10
Study risk of bias assessment	11	Specify the methods used to assess risk of bias in the included studies, including details of the tool(s) used, how many reviewers assessed each study and whether they worked independently, and if applicable, details of automation tools used in the process.	10
Effect measures	12	Specify for each outcome the effect measure(s) (e.g. risk ratio, mean difference) used in the synthesis or presentation of results.	10
Synthesis	13a	Describe the processes used to decide which studies were eligible for each synthesis (e.g. tabulating the study intervention characteristics and	11

Section and Topic	Item #	Checklist item	Location where item is reported
methods		comparing against the planned groups for each synthesis (item #5)).	
	13b	Describe any methods required to prepare the data for presentation or synthesis, such as handling of missing summary statistics, or data conversions.	10-11
	13c	Describe any methods used to tabulate or visually display results of individual studies and syntheses.	11
	13d	Describe any methods used to synthesize results and provide a rationale for the choice(s). If meta-analysis was performed, describe the model(s), method(s) to identify the presence and extent of statistical heterogeneity, and software package(s) used.	11
	13e	Describe any methods used to explore possible causes of heterogeneity among study results (e.g. subgroup analysis, meta-regression).	11
	13f	Describe any sensitivity analyses conducted to assess robustness of the synthesized results.	12
Reporting bias assessment	14	Describe any methods used to assess risk of bias due to missing results in a synthesis (arising from reporting biases).	10
Certainty assessment	15	Describe any methods used to assess certainty (or confidence) in the body of evidence for an outcome.	12
**RESULTS**			
Study selection	16a	Describe the results of the search and selection process, from the number of records identified in the search to the number of studies included in the review, ideally using a flow diagram.	12
	16b	Cite studies that might appear to meet the inclusion criteria, but which were excluded, and explain why they were excluded.	12
Study characteristics	17	Cite each included study and present its characteristics.	12
Risk of bias in studies	18	Present assessments of risk of bias for each included study.	14
Results of individual studies	19	For all outcomes, present, for each study: (a) summary statistics for each group (where appropriate) and (b) an effect estimate and its precision (e.g. confidence/credible interval), ideally using structured tables or plots.	15
Results of syntheses	20a	For each synthesis, briefly summarise the characteristics and risk of bias among contributing studies.	15
	20b	Present results of all statistical syntheses conducted. If meta-analysis was done, present for each the summary estimate and its precision (e.g. confidence/credible interval) and measures of statistical heterogeneity. If comparing groups, describe the direction of the effect.	15
	20c	Present results of all investigations of possible causes of heterogeneity among study results.	13
	20d	Present results of all sensitivity analyses conducted to assess the robustness of the synthesized results.	15
Reporting biases	21	Present assessments of risk of bias due to missing results (arising from reporting biases) for each synthesis assessed.	15
Certainty of evidence	22	Present assessments of certainty (or confidence) in the body of evidence for each outcome assessed.	16
**DISCUSSION**			

Section and Topic	Item #	Checklist item	Location where item is reported
Discussion	23a	Provide a general interpretation of the results in the context of other evidence.	16
	23b	Discuss any limitations of the evidence included in the review.	19
	23c	Discuss any limitations of the review processes used.	19
	23d	Discuss implications of the results for practice, policy, and future research.	20
**OTHER INFORMATION**			
Registration and protocol	24a	Provide registration information for the review, including register name and registration number, or state that the review was not registered.	3
	24b	Indicate where the review protocol can be accessed, or state that a protocol was not prepared.	3
	24c	Describe and explain any amendments to information provided at registration or in the protocol.	3
Support	25	Describe sources of financial or non-financial support for the review, and the role of the funders or sponsors in the review.	1
Competing interests	26	Declare any competing interests of review authors.	1
Availability of data, code and other materials	27	Report which of the following are publicly available and where they can be found: template data collection forms; data extracted from included studies; data used for all analyses; analytic code; any other materials used in the review.	Online appendix

From: Page MJ, McKenzie JE, Bossuyt PM, Boutron I, Hoffmann TC, Mulrow CD, et al. The PRISMA 2020 statement: an updated guideline for reporting systematic reviews. BMJ 2021;372:n71. doi: 10.1136/bmj.n71

For more information, visit: http://www.prisma-statement.org/
